# Impact of kidney function on the metabolome in the general population

**DOI:** 10.1371/journal.pone.0347652

**Published:** 2026-06-30

**Authors:** Johan Ljunggren, Mario Delgado-Velandia, Johan Sundström, Gunnar Engström, J. Gustav Smith, Johan Ärnlöv, Koen F. Dekkers, Sölve Elmståhl, Lars Lind, Maria K. Svensson

**Affiliations:** 1 Department of Medical Sciences, Uppsala University Hospital, Uppsala, Sweden; 2 Department of Clinical Sciences Malmö, Lund University, Malmö, Sweden; 3 Department of Cardiology, Clinical Sciences, Lund University and Skåne University hospital, Lund, Sweden; 4 Wallenberg Laboratory, Department of Molecular and Clinical Medicine, Institute of Medicine, Gothenburg University, Gothenburg, Sweden; 5 Depertment of Cardiology, Sahlgrenska University Hospital, Gothenburg, Sweden; 6 Wallenberg Center for Molecular Medicine and Lund University Diabetes Center, Lund University, Lund, Sweden; 7 Division of Family Medicine and Primary Care, Department of Neurobiology, Care Science and Society, Karolinska Institutet, Huddinge, Sweden; 8 School of Health and Social Studies, Dalarna University, Falun, Sweden; 9 Uppsala Clinical Research Center, Uppsala, Sweden; University of California Riverside, UNITED STATES OF AMERICA

## Abstract

**Background:**

A reduction in kidney function cause metabolites that normally are cleared by the kidney to accumulate. These accumulated metabolites could potentially link kidney function to an increased risk of cardiovascular disease.

**Aim:**

To assess the relationships between creatinine-based estimated glomerular filtration rate (eGFR) and the metabolome (791 endogenous metabolites) in the general population, as well as the cortisol to cortisone ratio.

**Patients and methods:**

Three population-based cohorts, Epihealth, PIVUS and POEM (total n = 3,444), were used with a discovery/validation approach. Metabolomics was measured by mass spectroscopy. eGFR was calculated using the 2021 CKD-EPI creatinine-based equation. Multivariable linear regression was used to assess the correlations between each metabolite concentration and eGFR, adjusting for sex, % fat mass, cardiovascular risk factors and medications. The metabolites significant in the linear regression were analyzed using MultiVariable Mendelian Randomization (MVMR), adjusting for diabetes and BMI.

**Results:**

Nighty-six metabolites were significantly associated to eGFR and had the same direction of association in both linear regression and MVMR. The vast majority of these associations were negative. Apart from creatinine, N-acetylalanine, N,N-dimethyl-pro-pro, N,N,N-trimethyl-alanylproline betaine (TMAP) were the top findings. Pathway enrichment analysis (Metaboloanalyst 6.0) found five significantly enriched pathways, two of which involved amino acid metabolism.

**Conclusions:**

In a general population with only mild to moderately reduced kidney function, several metabolites were associated with kidney function, including the cortisol to cortisone ratio. This finding is of interest since several of these metabolites might link reduced kidney function to an increased risk of cardiovascular disease.

## Background

The prevalence of chronic kidney disease (CKD) is increasing and currently afflicts about 10% of the general population [1,2]. This increase is in part explained by a growing prevalence of risk factors for CKD, the most important being older age, obesity, hypertension and diabetes. [1,2]

A reduction in glomerular filtration rate (GFR) causes metabolites that normally are cleared by the kidney to accumulate, via a reduction in plasma clearance. These changes are of interest since such metabolites might link CKD to an increased risk of cardiovascular disease (CVD) which is the leading cause of death in patients with CKD [3]. One potential mechanism could be endothelial damage [4–6]. Trimethylamine-N-oxide (TMAO) is a metabolite linked to both CKD and endothelial dysfunction where circulating levels have been shown to correlate with increased risk for myocardial infarction, stroke and death [7,8].

Previous studies utilizing metabolomics have found that several metabolites whose concentrations are associated with kidney function [1,5–7,9–14]. Examples of such metabolites include n-acetylated amino acids, which normally are metabolized in the kidney [12], metabolites involved in tryptophan metabolism [7], pseudouridine [1,6,9,11], erythronate [9], TMAO [7], and N,N,N-trimetyl-alanylproline betaine (TMAP) [13]. The majority of these metabolites increase when kidney function decline but not all studies are in agreement, with metabolites being significantly related to kidney function in some, but not all, studies [1,5–7,9–14].

Another metabolite of interest is cortisol and its ratio to cortisone. This ratio is skewed towards cortisol in CKD and an increased ratio has been shown to be a risk factor for CVD and mortality [15]. An increase in the ratio of cortisol to cortisone in CKD can be explained by several mechanisms, including an altered conversion between cortisol and cortisone and accumulation of cortisol metabolites [15].

Obesity is associated with the development of CKD [2] and has also been shown to induce profound changes in the metabolome [16]. Thus, fat mass affects kidney function, the metabolome, and also the estimation of kidney function (GFR) [17]. To our knowledge, previous studies on the association between kidney function and metabolites have not accounted for fat mass.

The aim of the present study was to assess the relationship between creatinine-based estimated glomerular filtration rate (eGFR) and the circulating metabolome (using 791 endogenous metabolites) in a general population with mild to moderately reduced kidney function. In addition, the relationship between creatinine-based eGFR and the cortisol to cortisone ratio was investigated. This was done in three population-based cohorts; Epihealth, POEM and PIVUS [18–21], employing a discovery/validation approach to minimize false positive findings. In addition, to obtain an estimate of the causal effect of kidney function on metabolite concentration in the blood, multivariable Mendelian randomization (MVMR) analysis was performed for relationships being significant in the observational setting.

## Subjects and methods

### Subjects

Three different population-based cohorts were used:

**EpiHealth.** The Epidemiology for Health study (EpiHealth) has been described elsewhere [18,19]. Starting in 2011, inhabitants in the cities of Uppsala and Malmö between the ages of 45 and 75 were randomly invited to participate in health survey. In 2018, a total of 25,000 patients had been recruited. The aim of the study was to investigate the connections between lifestyle factors, as well as genetic factors, and common diseases. Metabolomic data were analyzed from the first invited 2,342 subjects in the Uppsala sample. These individuals were used as the *discovery cohort* in this study.

**PIVUS.** The Prospective Study of the Vasculature in Uppsala Seniors (PIVUS) has also been described in detail elsewhere [18,21]. PIVUS was created with the goal to investigate if endothelial dysfunction implied increased cardiovascular risk. Starting in 2001, individuals in the city of Uppsala were recruited at the age of 70 and were then followed up at ages 75 and 80 years. Initially there were 1,016 individuals, out of which 50% were women. At the follow-up at age 80 (PIVUS80) 600 individuals attended. A DXA scan and an analysis of the metabolome were parts of the 80-year examination.

**POEM.** The Prospective investigation of Obesity, Energy and Metabolism (POEM) has also been described elsewhere [18,20]. In POEM, 502 individuals were recruited in the city of Uppsala between 2011 and 2016. All individuals were 50 years old and 50% were female. The aim of the study was to investigate how obesity affected cardiovascular disease.

In this study, the results from PIVUS80 and POEM were combined by meta-analysis and used as the *validation cohort*.

**SCAPIS** *(Swedish CArdioPulmonary Imaging Study)*

Between 2013 and 2018, a total of 30,154 men and women between the ages of 50 and 65 were recruited from six Swedish cites. The project was a collaboration between six universities in Sweden. [18] The participants underwent quantification of their cardiovascular risk factors, computed tomography coronary angiography, carotid artery ultrasound examination and further extensive imaging. For 4,985 participants in Uppsala and 3,978 participants in Malmö, metabolomic measurements on plasma were performed.

All studies were conducted with the informed consent from all participants, with approval from the responsible Ethics Committees in accordance with the principles of the Declaration of Helsinki (dnr 2010/402, 2011/045, 2009/057 and 2024-05807-01). The data were most recently accessed for research purposes between 2024-08-10 and 2025-06-27 and the authors had full access to information that could identify individual participants during that time period.

### Metabolomics

In all three study samples, non-targeted metabolomics (Metabolon inc., USA) was performed on plasma samples being stored at −80℃. Samples were prepared using the automated MicroLab STAR® system from Hamilton Company. Several internal standards were added prior to the first step in the extraction process for QC purposes. To remove protein, dissociate small molecules bound to protein or trapped in the precipitated protein matrix, and to recover chemically diverse metabolites, proteins were precipitated with methanol under vigorous shaking for 2 min (Glen Mills GenoGrinder 2000) followed by centrifugation. The resulting extract was divided into five fractions: two for analysis by two separate reverse phases (RP)/UPLC-MS/MS methods with positive ion mode electrospray ionization (ESI), one for analysis by RP/UPLC-MS/MS with negative ion mode ESI, one for analysis by hydrophilic interaction (HILIC)/UPLC-MS/MS with negative ion mode ESI, and one sample was reserved for backup. Only annotated, non-xenobiotic metabolites with a detection rate >75% in all samples were used in the analyses (n = 791). The values were normalized and given in arbitrary units.

Compounds were identified by comparison to library entries of purified standards or recurrent unknown entities. Metabolon maintains a library based on >3,000 authenticated standards that contains the retention time/index (RI), mass to charge ratio (m/z), and chromatographic data (including MS/MS spectral data) on all molecules present in the library.

Several types of controls were analyzed in concert with the experimental samples: a pooled matrix sample generated by taking a small volume of each experimental sample (or alternatively, use of a pool of well-characterized human plasma) served as a technical replicate throughout the data set; extracted water samples served as process blanks; and a cocktail of QC standards that were carefully chosen not to interfere with the measurement of endogenous compounds were spiked into every analyzed sample, allowed instrument performance monitoring and aided chromatographic alignment.

Instrument variability was 5% as determined by calculating the median relative standard deviation (RSD) for the internal standards that were added to each sample prior to injection into the mass spectrometers. Overall process variability was 7% as determined by calculating the median RSD for all endogenous metabolites (i.e., non-instrument standards) present in 100% of the Client Matrix samples, which are technical replicates of pooled client samples. In a published comparison between the 4 MS platforms used, the average laboratory coefficient of variation (CV) on the 4 platforms was between 9.3 and 11.5%, average inter-assay CV ranged from 9.9 to 12.6% and average intra-assay CV ranged from 5.7 to 6.9% [22].

### Kidney function

In both POEM and PIVUS80, plasma creatinine was measured by standard clinical laboratory methods. Creatinine was analyzed on Roche Cobas Pro instruments using an IDMS-calibrated enzymatic method for quantification. Results are then used to compute estimated glomerular filtration rates (eGFR). In Sweden, national IDMS calibration of creatinine assays was implemented in 2004 and all laboratories participate in external quality assurance programs for both creatinine and cystatin C (Equalis.se), ensuring consistent performance.

Such measurements were not performed in EpiHealth. However, creatinine was one of the metabolites measured in the metabolomic analysis, but the results are given on a relative scale. Since both PIVUS80 and POEM contained plasma creatinine measured by both metabolomics and standard methods, we fitted a linear regression model between the creatinine values obtained by the two different methods. The model obtained was *creatinine by standard methods* (µmol/L) = −28 + 107*metabolomics measured creatinine. This linear model was then used to estimate plasma creatinine in absolute values in EpiHealth. eGFR was thereafter calculated using the 2021 CKD-EPI creatinine-based equation [23] in all three cohorts.

### Dietary measurements

MiniMealQ, a comprehensive food frequency questionnaire, was a part of the internet-based questionnaire given to the participants of the EpiHealth study [24]. Including common Swedish beverages, foods and dishes, the MiniMealQ questionnaire covers the diet over the past few months. Depending on the food, either a five-level or eight-level ordinary scale was used to quantify the intake of the dietary items in the questionnaire. The five-level scale spanned from once to thrice monthly to more than seven times per week (vegetables, fruits, baked goods, and different dishes were estimated on this scale). The eight-level scale spanned from once to twice weekly to five or more times per day (example of products reported on this scale was dairy- and bread products). For alcohol, a six-level scale was used: spanning from never to four or more times weekly. From these data, the daily energy intake and protein intake were calculated.

### Fat mass and body weight

Fat mass and body weight was given from a weight scale that also calculates fat mass by mean of bioimpedance (Tanita, Tokyo, Japan) in all three cohorts used for the observational analysis. Fat mass percentage is fat mass divided by body weight.

### Genotyping

The extraction of DNA from the samples in SCAPIS was performed at the Karolinska Institutet Biobank. Utilizing Illumina Infinium Global Screening Array Multiple Disease version 3 and the SNP&SEQ Technology Platform, the samples were genotyped in 10 batches. For the unrelated samples, Principal component analysis (PCA) was performed and onto these components were projected all the samples. PLINK was used to make the PCA. All samples and markers with >2% missing data, with failed sex checks, with heterozygosity measures >3 standard deviations, with non-European ancestries, or with minor allele frequency < 0.1% and Hardy-Weinberg equilibrium P < 1x10^-8^ were removed as part of Pre-imputation quality control. Then, utilizing the “Pre-phasing and imputation with EAGLE2 + PBWT” pipeline, the Sanger Imputation Service was used to impute the SCAPIS genotype data to the HRCr 1.1.

A previous publication has described the genotyping of EpiHealth in detail [25]. In brief, from 400 µL EDTA whole blood, DNA was extracted and then dissolved in 145 µL 10 mmol/L Tris-HCl buffer (pH 8.0). The examination for quantity and purity was done by measuring absorbance at 230, 260 and 280 nm. The genotyping was performed with Illumina HumanCoreExome-12 v.1.0 BeadChip (Illumina, San Diego, CA, USA), which included 522,731 autosomal markers. Quality control was then performed, and after that 2378 samples remained, and their genotyping data was imputed up to the 1000 Genomes phase 3 (v5) reference panel. In the final dataset, about 12 million markers, with a minor allele count >= 1 were included.

#### Genome-wide association of metabolites in SCAPIS and EpiHealth.

Using 777 metabolites measured in both EpiHealth (N = 2,276) and SCAPIS (N = 8,133), applying linear mixed models in REGENIE, genome wide association tests were performed [26]. The genetic additive model used in the analysis was adjusted for genetic principal components 1–10, sex, age, age^2, Metabolon instrument batch in SCAPIS, and for genetic principal components 1–10, sex, age in EpiHealth. METAL version 2011-03-25 was used to perform a fixed-effects meta-analysis, based on inverse-variance weighting, of the association statistics of EpiHealth and SCAPIS [27].

### Genetic instruments of estimated glomerular filtration rate (eGFR), BMI and type 2 diabetes mellitus

We obtained summary-level GWAS statistics from a GWAS meta-analysis for serum creatinine eGFR (n = 1,004,040) including European-only data from the Chronic Kidney Disease Genetics Consortium and UK Biobank (PMID: 34272381). Likewise, European-only, summary-level GWAS statistics for BMI and T2DM were obtained from the UK Biobank (n = 405,010) [28], and the Diabetes Genetics Replication and Meta-analysis Consortium (DIAGRAM, n = 1,812,017) [29], respectively.

SNPs with a p-value < 5x10-8 for any of the three exposures were selected as instruments. In total, 173,848 instruments were found. Instruments were clumped remotely using the OpenGWAS server through the clump_data function (TwoSampleMR package, version 0.6.29), with default parameters: clumping p1 = 0.99, clumping R2 0.001, clumping window 10,000 kb, and European super-population as reference panel, leaving 860 instruments. Afterward, instruments with incomplete information for any of the exposures were removed, leaving 709 instruments. All these 709 SNPs were found in our in-house GWAS of plasma metabolites, but after data harmonization, removal of SNPs with incompatible alleles and palindromic SNPs with intermediate allele frequencies, 703 SNPs remained. The final number of instruments available for MR analyses was 190 for creatinine-based eGFR, 255 for body mass index (BMI) and 289 for type 2 diabetes mellitus (T2DM).

### Statistical methods

#### Observational analysis.

In order to make the distributions of the metabolites and eGFR standardized to the Z-scale and given a normal distribution, rank-based inverse normal transformation was used.

A discovery/validation approach was used with the EpiHealth cohort used as the discovery sample and a meta-analysis of POEM and PIVUS80 as the validation sample. Both the discovery and the validation analysis utilized multivariable linear regression. Multivariable linear regression models were fitted for each metabolite vs eGFR. The dependent variable was always the metabolite. In the discovery stage, eGFR, age, sex, non-fat mass, protein intake and energy intake were the independent variables. The first validation model (model 1) used eGFR and sex as independent variables. Since the age was 50 in all individuals in POEM and 80 in PIVUS80, age was not used as an independent variable. Validation model 2 used eGFR, sex and percent fat mass as independent variables, and validation model 3 used eGFR, sex, percent fat mass, systolic blood pressure, diabetes, number of years smoked, physical activity, use of statins and antihypertensive drugs as independent variables. The regression analyses of the validation cohorts were done separately. The results from the two cohorts were thereafter combined by an inverse-variance weighted (IVW) random effects meta-analysis.

For each model, participants with missing data in any of the variables were removed from the analysis for that model.

The p-values from the discovery cohort were adjusted using Benjamin-Hochberg to get the false discovery rate (FDR). The metabolites were deemed to be significant if they showed FDR < 0.05 in both discovery and validation for each of the models, respectively.

#### Mendelian randomization*.*

We performed a Multivariable Mendelian randomization (MVMR) on the association of creatinine-based eGFR with plasma metabolites adjusting for BMI and T2DM using SNP level summary data obtained from European populations with no known participant overlap between exposures and outcomes (Description of GWAS consortiums used is found in the supplemental materials, table in S5 Table). BMI and T2DM were used as covariates in the MVMR model, since BMI and T2DM were regarded as the most powerful confounders in the relationship between eGFR and the metabolites according to observational findings. Effect estimates were obtained using inverse variance weighting (IVW) and represent the effect of one standard deviation change in natural log transformed eGFR levels (ml/min/1.73 m^2^). In the supplemental materials, table in S6 Table, the instrumental strength (given as the F-value) is given for each SNP used in the MVMR. Only metabolites that were significant in the observational setting (model 3) were investigated with MVMR.

The resulting associations were corrected using FDR < 0.05. Only metabolites with the same direction of association in the MVMR and in the observational analysis were regarded as valid.

Analyses for the observational section were performed in R. Multivariable Mendelian randomization analyses were performed in Stata (version 16.1) using the mrmvivw command from the mrrobust package (version 0.3.0) (doi.org/10.1093/ije/dyy195).

### Pathway enrichment analysis

The metabolites that were significant and had the same direction in both validation model 3 and MVMR were used for a pathway enrichment analysis using MetaboAnalyst (https://www.metaboanalyst.ca/MetaboAnalyst/ModuleView.xhtml). Here, a Hypergeometric test with relative betweenness centrality was used. The selected library was the *Homo sapiens* KEGG library. The significance level was set to a nominal p < 0.05.

## Results

### Clinical and biochemical characteristics of study participants

The basic clinical and biochemical characteristics of study participants in EpiHealth, PIVUS80, and POEM are presented in Table 1. The major difference between the cohorts was age. In Epihealth mean age of the participants was 61 years, while in PIVUS80 all participants were 80 and in POEM 50 years. Study participants of higher age had higher systolic blood pressure, were more often treated with statins and antihypertensive medication, and had a lower eGFR. The average eGFR for each cohort was within two standard deviations of the expected value based on their average age [30,31].

**Table 1:** Clinical and biochemical characteristics of study participants in EpiHealth, PIVUS80 and POEM.

	EpiHealth	PIVUS80	POEM
n	2342	600	502
Age (years)	61.1 (8.4)	80	50
Female sex (%)	49.5	49.5	49.6
Systolic blood pressure (mmHg)	134.6 (17.1)	146.8 (19.4)	125.6 (16.4)
Diastolic blood pressure (mmHg)	83.4 (9.4)	73.8 (9)	77 (10.2)
HDL-cholesterol (mmol/L)	1.51 (0.4)	1.38 (0.4)	1.39 (0.4)
Triglycerides (mmol/L)	1.29 (0.8)	1.24 (0.6)	1.19 (0.9)
BMI (kg/m^2^)	26.5 (3.8)	26.9 (4.5)	26.5 (4.3)
Waist circumference (cm)	92.6 (11.8)	96.4 (11.8)	92.6 (11.5)
Fat mass (%)	23.9 (8.3)	32.7 (8.1)	28.0 (8.5)
Fasting glucose (mmol/L)	5.99 (1.0)	5.28 (1.4)	5.08 (0.9)
Diabetes medication^a^ (%)	2.5	10.5	1.4
Antihypertensive medication (%)	22.2	59.8	8.4
Statin use (%)	---	32.8	3.8
Exercise^b^	3.08 (0.95)	4.05 (2.5) low	3.43 (2.6) low
		0.43 (0.9) high	1.62 (1.6) high
Education (%) <10 years	21.4		7.4
10–12 years	28.5		43.7
>12 years	50.1		48.9
Current smoker (%)	---	3.04	9.82
eGFR (ml/min^/^1.73 m^2^)	85.2 (13.1)	71.6 (14.2)	1 (11.9)

Data is presented as mean (sd) or % of study participants. ^a^For PIVUS80 and POEM. Diabetes medication is defined as oral anti-diabetic medication or insulin. (or both). ^b^Epihealth had a single measure for physical activity. ranging from 1 to 5. PIVUS80 and POEM have separate categories for low intensity and high intensity physical activity. measured in number of 30-minute sessions per week of low and high intensity exercise respectively. eGFR; creatinine-based estimated glomerular filtration rate.

Because of missing data, 53 participants were removed from the analysis of model 2 and 176 participants were removed from model 3. Thus, the number of participants were 1102 in model 1, 1049 in model 2, and 926 in model 3.

### Linear regression between metabolites and kidney function (eGFR)

**Fig 1:**
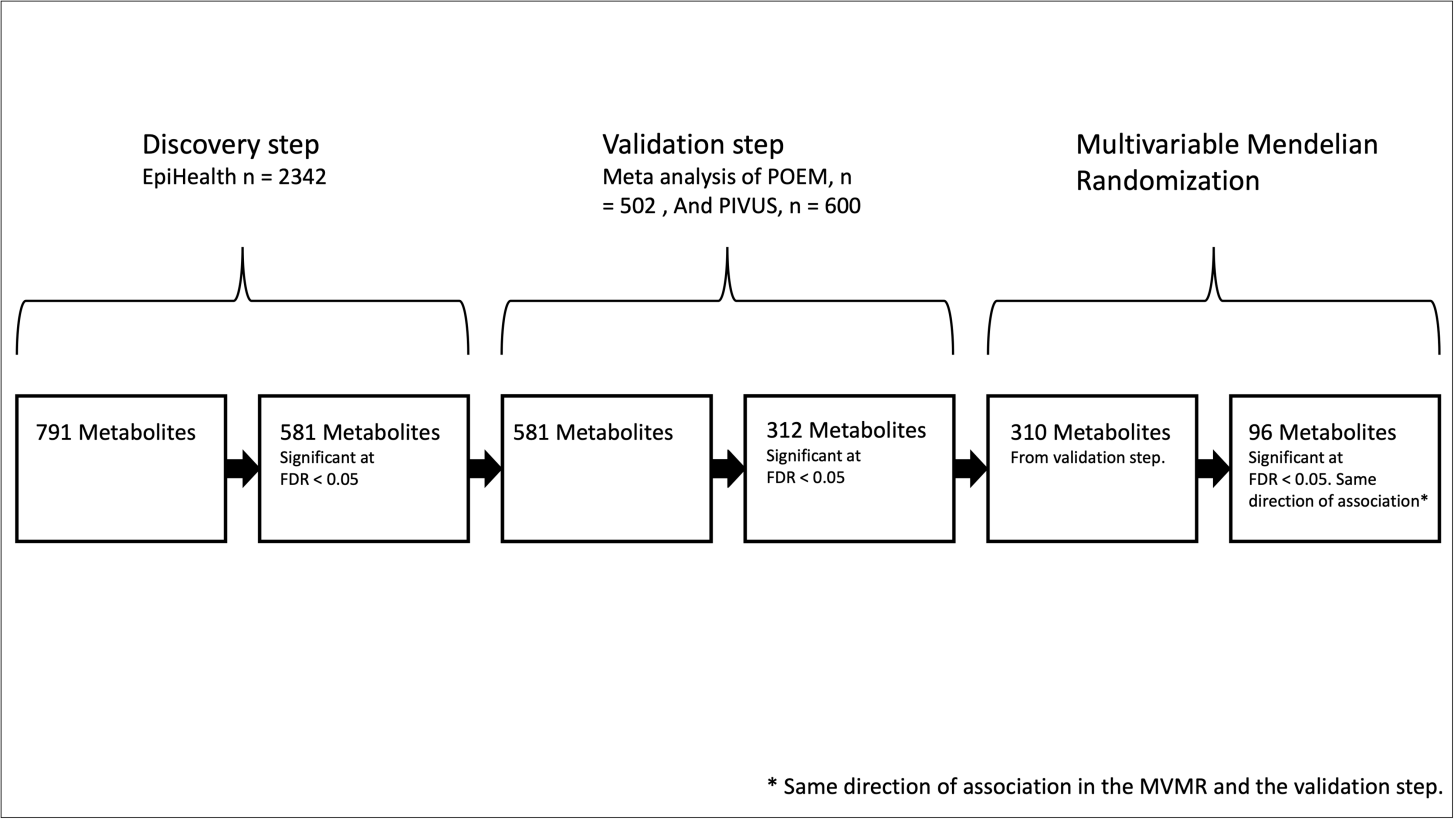
A diagram of the workflow.

The workflow of this paper is described in Fig 1. Out of 791 metabolites analyzed, 254 had a significant regression coefficient for the eGFR variable at FDR < 0.05 in model 1with eGFR and sex as independent variables. Six of these coefficients were positive while the remaining were negative (see supplemental materials, table in S1 Table). In Table 2, the 15 metabolites with the largest negative coefficients are shown. These were creatinine, N,N-dimethyl-pro-pro, N,N,N-trimethyl-alanylproline betaine (TMAP), N-acetylalanine, N-acetylserine, N-formylmethionine, pseudouridine, 1-methylhistidine, 2,3-dihydroxy-5-methylthio-4-pentenoate (DMTPA)*, 3-(3-amino-3-carboxypropyl)uridine*, hydroxyasparagine**, N-acetylthreonine, N6-carbamoylthreonyladenosine, C-glycosyltryptophan, and erythronate. In addition, the cortisol to cortisone ratio had, for the eGFR variable, a regression coefficient of −0.25, FDR = 5.69*10^−4^. The metabolites with a positive regression coefficient were creatine, alpha-ketobutyrate, 1-palmitoyl-2-palmitoleoyl-GPC (16:0/16:1)*, 1-palmitoyl-2-oleoyl-GPC (16:0/18:1), 1-stearoyl-2-oleoyl-GPC (18:0/18:1), and 2-aminobutyrate (see Table 2). The metabolite with the largest negative coefficient was creatinine, and the one with the largest positive coefficient was creatine.

**Table 2:** The 15 metabolites with the largest negative regression coefficients and the metabolites with positive regression coefficients with creatinine-based eGFR in the combined results from the validation cohorts analyzed with model 1.

Metabolite	beta1	Std error	P-value	FDR
Top 15 negatively associated metabolites				
creatinine	−0.7573651	0.03641826	4.6752E-96	2.7163E-93
N,N-dimethyl-pro-pro	−0.6249709	0.07139888	2.0733E-18	8.6042E-17
N,N,N-trimethyl-alanylproline betaine (TMAP)	−0.6242499	0.11298204	3.2909E-08	2.897E-07
N-acetylalanine	−0.6066361	0.09714994	4.2562E-10	5.1518E-09
N-acetylserine	−0.5700514	0.08445955	1.4846E-11	2.1563E-10
N-formylmethionine	−0.5612657	0.07536216	9.5068E-14	1.9727E-12
pseudouridine	−0.5597386	0.09459957	3.2802E-09	3.4032E-08
1-methylhistidine	−0.5579634	0.10509126	1.1003E-07	9.1328E-07
2,3-dihydroxy-5-methylthio-4-pentenoate (DMTPA)*	−0.5536878	0.14289661	0.00010674	0.00045599
3-(3-amino-3-carboxypropyl)uridine*	−0.5481795	0.13410995	4.3597E-05	0.0002165
hydroxyasparagine**	−0.5367139	0.15292835	0.00044882	0.00165041
N-acetylthreonine	−0.5356437	0.11688187	4.5884E-06	2.9295E-05
N6-carbamoylthreonyladenosine	−0.5314072	0.09144559	6.2032E-09	6.125E-08
C-glycosyltryptophan	−0.5291234	0.11587068	4.9594E-06	3.1319E-05
erythronate*	−0.503233	0.15400183	0.00108422	0.00353895
Positively associated metabolites				
creatine	0.18385483	0.02631205	2.7988E-12	4.3948E-11
alpha-ketobutyrate	0.12620061	0.03575211	0.00041575	0.00155839
1-palmitoyl-2-palmitoleoyl-GPC (16:0/16:1)*	0.09885734	0.02960288	0.00083944	0.00285214
1-palmitoyl-2-oleoyl-GPC (16:0/18:1)	0.07842389	0.03004562	0.00904997	0.02373375
1-stearoyl-2-oleoyl-GPC (18:0/18:1)	0.07817689	0.02971791	0.00852262	0.02261023
2-aminobutyrate	0.07298369	0.03019494	0.01564542	0.03803343

Model 1 had eGFR and sex as independent variables and the metabolite measurement as dependent variable.

In model 2, further adjusting for fat mass (%), 270 metabolites had a significant regression coefficient for eGFR, at FDR < 0.05, seven were positive and the rest negative. 29 metabolites were significant in model 2 but not in model 1, while 13 metabolites were significant in model 1, but not in model 2. In S2 Table, all metabolites being significant in model 2 are shown. In Table 3, the metabolites that were significant in model 2 but not in model 1 are listed. The metabolites with a positive regression coefficient in model 2 were creatine, alpha-ketobutyrate, aspartate, 1-palmitoyl-2-palmitoleoyl-GPC (16:0/16:1)*, 1-stearoyl-2-oleoyl-GPC (18:0/18:1), 1-palmitoyl-2-oleoyl-GPC (16:0/18:1), and 1,2-dipalmitoyl-GPC (16:0/16:0), se Table 4. Also, in model 2, the cortisol to cortisone ratio was strongly associated to eGFR (regression coefficient −0.24, FDR = 3.85*10^−4^). In model 2, the largest positive coefficient was for creatine. A different regression model was fit with independent variables eGFR, sex and body fat in kg instead of percent fat mass. The results from this analysis were similar to those of model 2 (data not shown).

**Table 3:** The 29 metabolites that were significant in model 2 but not in model 1.

chemical_name	beta1	Std error	p_value	FDR
1-linoleoylglycerol (18:2)	−0.0738722	0.03081641	0.01652223	0.03663899
1-methylnicotinamide	−0.1319184	0.04836613	0.00638172	0.01591322
1-oleoyl-2-arachidonoyl-GPE (18:1/20:4)*	−0.115112	0.05031967	0.02215999	0.04768501
1,2-dipalmitoyl-GPC (16:0/16:0)	0.08916344	0.03308625	0.00704141	0.01709704
2-O-methylascorbic acid	−0.3269372	0.09697129	0.00074764	0.00237367
4-methoxyphenol sulfate	−0.0994942	0.02925365	0.00067119	0.00215447
5-(galactosylhydroxy)-L-lysine	−0.3148728	0.13415613	0.01892208	0.0413298
5-methylthioadenosine (MTA)	−0.3650754	0.13360784	0.00628671	0.01574388
5-methyluridine (ribothymidine)	−0.1440362	0.04842321	0.00293439	0.00800413
5,6-dihydrothymine	−0.1650331	0.05129419	0.00129365	0.00391464
aconitate [cis or trans]	−0.3118634	0.10998699	0.00457601	0.011764
andro steroid monosulfate C19H28O6S (1)*	−0.0796835	0.02959464	0.00709189	0.01709704
aspartate	0.11675517	0.04409743	0.008105	0.0192992
beta-hydroxyisovalerate	−0.1525644	0.05933759	0.01013687	0.02367362
cis-3,4-methyleneheptanoate	−0.0873319	0.03122068	0.00515406	0.01307645
cis-3,4-methyleneheptanoylcarnitine	−0.1770717	0.07447993	0.01743318	0.03836619
cystathionine	−0.2786842	0.11560378	0.01592262	0.03558093
dihydroorotate	−0.1319963	0.05562063	0.0176369	0.03866807
dodecanedioate (C12-DC)	−0.1223544	0.03101339	7.973E-05	0.00032198
gamma-glutamylalanine	−0.0936413	0.03109047	0.0025962	0.00714878
gamma-glutamylleucine	−0.2841082	0.0938506	0.00246799	0.00686077
kynurenate	−0.4088331	0.16942469	0.01581893	0.0354857
N-acetylcitrulline	−0.1696292	0.06664371	0.01091792	0.02517186
N,N,N-trimethyl-5-aminovalerate	−0.1720409	0.0587181	0.00339019	0.009119
oleoyl-linoleoyl-glycerol (18:1/18:2) [2]	−0.1482695	0.0475216	0.00180824	0.00525294
orotate	−0.1837985	0.07569771	0.01517989	0.03418416
S-adenosylhomocysteine (SAH)	−0.4409476	0.17668339	0.01257111	0.02864241
succinylcarnitine (C4-DC)	−0.3453332	0.12058823	0.00418673	0.01095715
urate	−0.369377	0.12215221	0.00249533	0.00690374

Model 1 had eGFR and sex as independent variables.

Model 2 had eGFR. sex and percent fat mass as independent variables. Both had the metabolite measurements as the dependent variables.

**Table 4:** The metabolites with a positive regression coefficient with creatinine-based eGFR in the combined results from the validation cohorts using model 2.

Metabolite	beta1	Std error	p-value	FDR
creatine	0.17634532	0.03111339	1.4462E-08	1.3552E-07
alpha-ketobutyrate	0.1223864	0.03055362	6.1853E-05	0.00026041
aspartate	0.11675517	0.04409743	0.008105	0.0192992
1-palmitoyl-2-palmitoleoyl-GPC (16:0/16:1)*	0.10947658	0.03084189	0.00038581	0.00137518
1-stearoyl-2-oleoyl-GPC (18:0/18:1)	0.10144363	0.03080902	0.00099244	0.00305084
1-palmitoyl-2-oleoyl-GPC (16:0/18:1)	0.08925424	0.03111438	0.00412313	0.01084496
1,2-dipalmitoyl-GPC (16:0/16:0)	0.08916344	0.03308625	0.00704141	0.01709704

Model 2 had eGFR. sex and percent fat mass as independent variables and the metabolite measurement as dependent variable.

**Fig 2:**
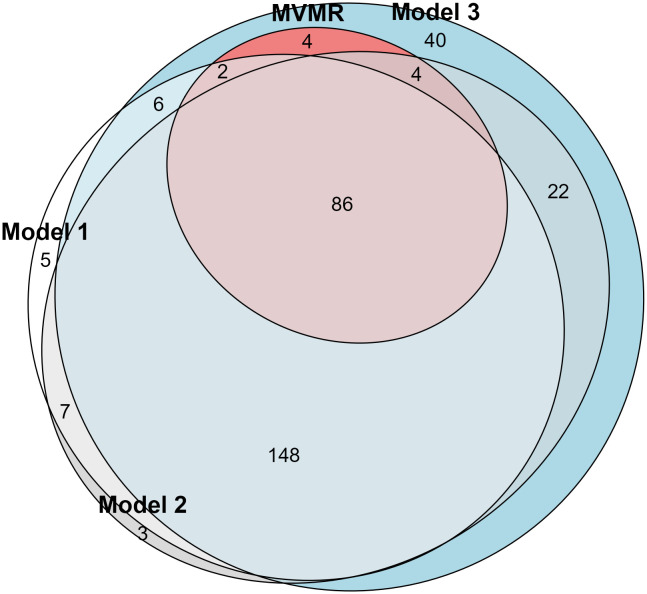
A Venn diagram depicting the number of metabolites that are significant in model 1, 2, 3 and the MultiVariable Mendelian Randomization (MVMR) analysis.

In model 3, also adjusting for cardiovascular risk factors and medications, 312 metabolites had a significant coefficient for eGFR. Six of these were positive while the rest, 306, were negative. Compared with model 1, there were 70 metabolites being significant in model 3 but not in model 1, and 12 significant metabolites in model 1 that were not significant in model 3. Compared with model 2, there are 52 metabolites that are significant in model 3 that are not significant in model 2 and 10 that are significant in model 2 but not in model 3. A Venn diagram over the significant metabolites in each model is shown in Fig 2. All significant metabolites in model 3 are presented in S3 Table. The 15 metabolites with the largest negative regression coefficients in model 3 were creatinine, N-acetylalanine, N,N-dimethyl-pro-pro, N,N,N-trimethyl-alanylproline betaine (TMAP), N-acetylserine, pseudouridine, 3-(3-amino-3-carboxypropyl)uridine*, N-formylmethionine, 2,3-dihydroxy-5-methylthio-4-pentenoate (DMTPA)*, hydroxyasparagine**, C-glycosyltryptophan, N-acetylthreonine, N6-carbamoylthreonyladenosine, 1-methylhistidine, and 5,6-dihydrouridine. The metabolites in model 3 with a positive regression coefficient for eGFR were creatine, glutamate, aspartate, alpha-ketobutyrate, mannose, and S-methylcysteine, see Table 5. The cortisol to cortisone ratio had a regression coefficient for eGFR of approximately −0.26 and FDR = 4.79*10^−3^ in model 3. The metabolite with the largest negative regression coefficient for eGFR in model 3 was creatinine, and the one with largest positive coefficient was creatine.

**Table 5:** The 15 metabolites with the largest negative regression coefficients and all metabolites with positive regression coefficients with creatinine-based eGFR in the combined results from the validation cohorts using model 3.

chemical_name	beta1	Std error	p-value	FDR
Top 15 negatively associated metabolites				
creatinine	−0.7645856	0.02638249	1.149E-184	6.674E-182
N-acetylalanine	−0.6648561	0.06108168	1.3634E-27	2.6405E-25
N,N-dimethyl-pro-pro	−0.6490193	0.07027158	2.5619E-20	1.4885E-18
N,N,N-trimethyl-alanylproline betaine (TMAP)	−0.639178	0.10461797	9.9857E-10	1.0178E-08
N-acetylserine	−0.6229853	0.06634847	6.0231E-21	3.8882E-19
pseudouridine	−0.6001668	0.09429699	1.9574E-10	2.187E-09
3-(3-amino-3-carboxypropyl)uridine*	−0.5976384	0.10003677	2.3124E-09	2.2392E-08
N-formylmethionine	−0.5956084	0.06932014	8.5363E-18	3.0998E-16
2,3-dihydroxy-5-methylthio-4-pentenoate (DMTPA)*	−0.5904982	0.08802218	1.9661E-11	2.6566E-10
hydroxyasparagine**	−0.5849247	0.08983376	7.4558E-11	8.8404E-10
C-glycosyltryptophan	−0.5848724	0.09874997	3.1658E-09	3.0153E-08
N-acetylthreonine	−0.5696709	0.11638718	9.8497E-07	5.851E-06
N6-carbamoylthreonyladenosine	−0.5648694	0.05568699	3.5362E-24	4.1091E-22
1-methylhistidine	−0.5560535	0.10116205	3.8705E-08	3.1233E-07
5,6-dihydrouridine	−0.5420584	0.09811907	3.3043E-08	2.7426E-07
Positively associated metabolites				
creatine	0.16889905	0.04754445	0.00038167	0.00118583
glutamate	0.15788216	0.03210476	8.7559E-07	5.3549E-06
aspartate	0.14018106	0.03271593	1.8291E-05	8.0507E-05
alpha-ketobutyrate	0.13208973	0.03399527	0.00010211	0.00036395
mannose	0.12627885	0.03027126	3.025E-05	0.00012644
S-methylcysteine	0.07778974	0.03370799	0.02101285	0.04055968

Model 3 had eGFR, Sex, percent fat mass, systolic blood pressure, Diabetes, number of years smoked, physical activity, use of statins, and medicine against hypertension as independent variables, and the metabolite measurement as dependent variable.

### Multivariable Mendelian randomization (MVMR)

MVMR analysis was performed for 310 out of the 312 significant metabolites in model 3. Out of these 98 were significant at FDR < 0.05 and out of those, 96 had the same direction of association in MVMR as in the observational analysis. The only metabolite with a positive direction of association was for creatine. The 15 metabolites with the largest negative coefficients in the MVMR analysis were: creatinine, 1-methylhistidine, N-acetylalanine, hydroxyasparagine**, N,N,N-trimethyl-alanylproline betaine (TMAP), homocitrulline, N,N-dimethyl-pro-pro, 3-(3-amino-3-carboxypropyl)uridine*, methionine sulfone, 2,3-dihydroxy-5-methylthio-4-pentenoate (DMTPA)*, 3-hydroxy-3-methylglutarate, N-acetylserine, N6-carbamoylthreonyladenosine, pseudouridine, and deoxycarnitine, see Table 6. The complete results are found in the table in S4 Table.

**Table 6:** The 15 metabolites with the largest negative association coefficients and the metabolites with positive association coefficient in the Multivariable Mendelian randomization (MVMR) analysis.

Metabolite	Beta	Std error	p-value	FDR
Top 15 negatively associated metabolites				
creatinine	−5.048728	0.51905334	2.31769E-22	7.1385E-20
1-methylhistidine	−4.5768671	0.61653656	1.14054E-13	1.7564E-11
N-acetylalanine	−3.8759553	0.61426228	2.79152E-10	2.1046E-08
hydroxyasparagine**	−3.5875652	0.59472579	1.61641E-09	8.2976E-08
N,N,N-trimethyl-alanylproline betaine (TMAP)	−3.5861537	0.55028558	7.17772E-11	7.3691E-09
homocitrulline	−3.5811503	0.61766142	6.71433E-09	2.585E-07
N,N-dimethyl-pro-pro	−3.5449772	0.5646134	3.41653E-10	2.1046E-08
3-(3-amino-3-carboxypropyl)uridine*	−3.5124848	0.61360866	1.03857E-08	3.5542E-07
methionine sulfone	−3.4069366	0.56831557	2.03739E-09	8.9645E-08
2,3-dihydroxy-5-methylthio-4-pentenoate (DMTPA)*	−3.3234906	0.58596063	1.4125E-08	4.3505E-07
3-hydroxy-3-methylglutarate	−3.2342403	0.61267543	1.29976E-07	3.336E-06
N-acetylserine	−3.0929105	0.61742377	5.46055E-07	1.2013E-05
N6-carbamoylthreonyladenosine	−3.0511811	0.59428382	2.83324E-07	6.7126E-06
pseudouridine	−3.0462642	0.61432737	7.09671E-07	1.3661E-05
deoxycarnitine	−3.0119729	0.53555721	1.86587E-08	5.2244E-07
Positively associated metabolites				
creatine	1.4094682	0.56022137	0.011872455	0.03898153

The MVMR analysis was performed for the metabolites that were significant in Model 3 of the observational analysis, and adjusted for BMI and T2DM.

### Pathway enrichment analysis (KEGG)

The 96 metabolites that were significant and had the same direction of association in the MVMR and observational analysis were used in a pathway enrichment analysis. In total, there were five significant pathways at p < 0.05, as shown in table 7. Of these five significantly enriched pathways, two involved amino acid metabolism.

**Table 7:** The metabolite pathways shown to be significant in the pathway enrichment analysis.

Pathway	Total	Expected	Hits	p-value	Impact
Ascorbate and aldarate metabolism	9	0.10176	2	0.0041493	0.76191
Arginine biosynthesis	14	0.15829	2	0.010142	0.23936
Pentose and glucuronate interconversions	19	0.21482	2	0.018432	0.15663
One carbon pool by folate	26	0.29397	2	0.033431	0.08884
Lysine degradation	30	0.3392	2	0.04357	0.00204

Metabolites used in the analysis were all significant metabolites from the combined results of the validation cohorts using model 3. The significance level was unadjusted p-value <0.05. MetaboAnalyst 6.0 was used. Total; the total number of metabolites in the KEGG pathway. Expected; the expected number of metabolites belonging to that pathway if not enriched. Hits; the number of metabolites used in the analysis that belonged to that pathway. Impact; a measure that combines results from pathway enrichment analysis and measures of centrality in the pathway of the found metabolites [32].

## Discussion

This is to our knowledge the largest comprehensive study exploring the relationship between kidney function (creatinine-based eGFR) and the metabolome in a general population with only mild to moderately reduced kidney function. In this study, we applied a robust two-stage design using discovery and validation cohorts, coupled with stringent multiplicity testing, to identify metabolites and explore their association with creatinine-based eGFR. This approach not only minimizes the risk of false discoveries, but also provides insights into the underlying biological processes by focusing on individual metabolites and their pathways. The number of validated metabolites found to be related to eGFR were 254 when adjusting for age and sex; 270 if fat mass was added, and 312 when adjustment also was done for traditional cardiovascular risk factors and medications. Mendelian randomization analysis revealed that 96 of these metabolites were significant and had the same direction of association as in the observational analysis.

Most of these metabolites were inversely associated with eGFR. This is not unexpected since the kidneys play a major part in the plasma clearance of metabolites. Neither was it surprising that the metabolite with the largest regression coefficient to eGFR estimated using creatinine was creatinine in itself, in this case thus serving as a positive control.

Many significant metabolites in this study have been described previously in relation to kidney function. For example, c-mannosyltryptophan, erythronate, pseudouridine, and n-acetylated amino acids were found by several other groups [1,6,7,9,11,12]. Several amino n-acetylated amino acids were associated with eGFR. This is also expected since metabolism of these compounds occurs mainly in the kidney [33]. 1-palmitoyl-2-oleoyl-GPC (16:0/18:1) and 1-stearoyl-2-oleoyl-GPC (18:0/18:1) are significant and positive in model 1 and model 2 but not in model 3.The cortisol to cortisone ratio was negatively related to eGFR in all linear regression models. This suggests that the activity of 11β-HSD type 2, an enzyme that converts active 11β-hydroxyglucocorticoids (cortisol) to their inactive 11-keto forms (cortisone) and thereby fine-tuning the activation of mineralocorticoid and glucocorticoid receptors, is reduced and correlated with kidney function, which also has been shown in previous studies [34–35]. A recent publication showed that a decline in 11β-HSD2 activity was observed with progressively lower eGFR in individuals spanning a wide spectrum of kidney function, including those with apparently normal kidney function [36]. This is in line with the findings in this present study. Cortisol in itself was not significantly associated with eGFR in any validation model. However, the average eGFR in these cohorts were relatively high [30,31], and it is possible that changes in cortisol itself are only detectable at lower values of eGFR.

The population-based cohorts used in this study displayed a higher mean eGFR than in most previous studies, supporting that these changes are present already in a general population with only mild to moderately reduced kidney function.

A previous study investigating metabolites associated with an increased risk of incident CVD in EpiHealth showed that 37 metabolites were correlated to an increased risk of CVD, ten of which were significantly related to eGFR in the multiple-adjusted model in the present study. Some were strongly associated to eGFR with three of the metabolites being in the top 15 most negatively associated metabolites in the MVMR analysis of the metabolites significant in both observational and MVMR: N-acetylalanine, N-acetylserine, and N6-carbamoylthreonyladenosine. Out of these, all had a hazard ratio below above 1 for incident CVD [37]. In a study by Pietzner et.al [38], they investigate the correlation between the metabolome and 27 non-comunicable diseases and mortality. Comparing the significant metabolites in model 3 and MVMR with the significant metabolites found by Pietzner et.al. for the outcomes: kidney disease, CVD and mortality show that 42 of the significant metabolites in model 3 were related to these outcomes. Of these were 21 related to kidney disease, 31 to arterial CVD (atrial fibrillation, heart failure, cerebrovascular infarction, ischemic heart disease, peripheral arterial disease and abdominal aorta aneurysm) and 40 with mortality. The majority of these were associated with both increased hazard ratio of the conditions (kidney disease, CVD and mortality) and inversely related to eGFR.

Future studies have to disentangle if some of these metabolites are involved in the known relationship between CKD and future CVD [39].

The pathway enrichment analysis was exploratory in nature. It found 5 enriched pathways. 2 of those pathways involve amino acid synthesis or metabolism as would be expected, but also pentose and glucoronate intraconversions, as well as ascorbate and aldarate metabolism were amongthe enriched pathways. The ascorbate and aldarate metabolism pathway, and the lysine degradation pathway have previously been shown to be associated to CKD progression [39]. When considering the link to CVD, the lysine degradation pathway has been shown to be enriched in the urine of patients with unstable angina [40]. Other studies of the metabolome that included pathway enrichment analysisin patients with CVD did not find these pathways to be enriched [37,41,42].

The ascorbate and aldarate metabolism pathway is vitamin C metabolism. Patients with CKD have lower vitamin C, primarily due to decreased intake caused by decreased appetite and dietary restrictions, as well as losses from dialysis treatment [43]. These causes are unlikely to be the reasons that these pathways were significantly enriched in this study, which investigated the general population. One function of vitamin C is as an antioxidant. CKD is associated with oxidative stress and vitamin C might counteract part of this effect [43]. However, in patients with decreased GFR, oxalate formed from breakdown of vitamin C can accumulate in the blood, which may cause cardiovascular damage [43].

Previous research has shown that the gut microbiota plays a role in plasma metabolomics [44]. Koen F. Dekkers and colleagues have developed the GUTSY atlas, which for example, quantifies the proportion of variance in specific plasma metabolites attributable to microbiome variations, calculated using nested 10-fold cross-validated ridge regressions. Using those results and applying them to the 96 metabolites that were significant and had the same direction of association in both model 3 and the MVMR analysis in this study showed that the variances of 91 metabolites were influenced by the microbiome. However, for most of these metabolites, the effect of the microbiome was small, and only 12 had ≥ 5% of their variance explained by the microbiome.

Creatinine levels were measured by mass spectroscopy as a part of the metabolomic evaluation in the EpiHealth cohort and were reported on a relative scale only. Therefore, the mean value for eGFR in the Epihealth study was estimated by a linear regression model using data from the POEM and PIVUS studies, in which creatinine was measured by both clinical chemistry methods (providing absolute values) and by the same mass spectroscopy method as in the EpiHealth cohort. The correlation between the clinical chemistry and metabolomic methods in PIVUS and POEM was high (0.92–0.93). It is important to notethat clinical chemistry measured creatinine was used in the validation step, thereby mitigating any error introduced by the mass spectroscopy measured creatinine in the discovery step.

Creatinine based eGFR was used because it is the dominantly used measurement in the clinical setting. Furthermore, other compounds that are used to estimate eGFR, such as Cystatin C, were not measured in the cohorts used in this study.

The observed changes in metabolite concentrations seen in this study are likely a result of decreased clearance rather than dysregulation of any specific metabolic pathway. The reason for this is that the eGFR values of the participants in this study are normal or only slightly reduced. Dysregulation of more specific metabolic pathways might emerge later in the course of CKD progression.

The metabolomic measurements, namely UPLC-MS/MS, was performed by a commercial company (Metabolon Inc, U.S.A). Therefore, some details of the metabolic measurement are trade secrets of the company and thushus, a fully detailed description is not publicly available.

The major strength of the present study is the use of a metabolomic platform that made it possible to evaluate a wide range of non-xenobiotic metabolites in these large population-based samples. This ensures high-quality data with accuracy and reliability of the metabolomic measurements across cohorts. In addition, the large sample sizes used in both the discovery and validation cohorts enhance the statistical power of the study, allowing for the detection of robust associations between eGFR and metabolites of interest. The use of population-based cohorts adds to the study’s external validity, improving the representativeness of the findings across different populations. We should however acknowledge that we have studied almost exclusively individuals with European ancestry, so future studies in other ethnic groups are warranted. Another limitation is that in the discovery cohort, creatinine was not measured using standard clinical chemistry methods, which could potentially introduce a small variability or bias in the assessment of kidney function. Additionally, the lack of data on most medications used by the participants represents a limitation.

In conclusion, in this study, to our knowledge, the largest comprehensive study of the relationship between kidney function and the metabolome in a general population with mild to moderately reduced kidney function, identified several cardiovascular disease-related metabolites related to CVD, including the cortisol to cortisone ratio, as being associated with kidney function. For most of these metabolites, the effect of the microbiome was small.

## Supporting information

S1 Table Complete results model 1. (XLSX)

S2 Table Complete results model 2. (XLSX)

S3 Table Complete results model 3. (XLSX)

S4 Table Complete results MVMR. (XLSX)

S5 Table Description of GWAS consortiums used in the MVMR analysis. (XLSX)

S6 Table The instrumental strength for the SNPs used in the MVMR analysis. (XLSX)
