# Supplementary material for: Impact of kidney function on the metabolome in the general population
Source: PLoS One. 2026 Jun 30;21(6):e0347652. doi: 10.1371/journal.pone.0347652 (PMC13318015; doi:10.1371/journal.pone.0347652)
Supplement: Attachment — Submitted filename: Reviewer comments.docx [file pone.0347652.s007.docx]

Title: Impact of kidney function on the metabolome in the general population

Manuscript number: PONE-D-25-35075

Reviewer comments:

The current manuscript provides a timely and comprehensive metabolomic investigation into the associations between estimated glomerular filtration rate (eGFR) and a wide array of endogenous metabolites in a large, general population cohort. The research directly addresses an important pathophysiological hypothesis—that retained metabolites may mediate the well-established link between chronic kidney disease and cardiovascular risk.

However, there are following questions that should be answered/clarified by the author and updated in the manuscript that will help in overall quality of the manuscript.

1. Line 108 > please mention degree Celsius or degree Fahrenheit.
2. Line 109 > The authors have mentioned the addition of internal standards in plasma samples before extraction. However, the author’s should mention what are the internal standards, their name, their concentration added and how they were useful in the analysis. These details are lacking in the entire manuscript. Also please add them in the results section.
3. In subjects and methods, the authors have just mentioned the use of reverse phase UPLC-MS/MS and HILIC analytical method for metabolomic study. However, they haven’t mentioned any details, The authors are advised to add details such as column used, mobile phase used, its elution profile, the mass spectrometry parameters, what instrument was the samples analyzed upon. These details are very crucial in determining the overall process of metabolomic analysis, be it targeted or untargeted.
4. The authors are advised to provide step by step procedure for conducting experimental metabolomic analysis.
5. The authors are also advised to present steps of identification of metabolites from untargeted to targeted, their selection and annotation criteria, and software used in this process.
6. The authors have mentioned the samples from all three cohorts were analyzed in same fashion and calibrated for being comparable. Please clarify how they were calibrated.
7. Multivariate analysis is mentioned in the abstract. However, what multivariate analysis, like PCA, or PLSDA or any other analysis was used and their results are missing in the manuscript. Please clarify how the multivariate analysis was used.
8. Please explain in detail what the validation models are and clarify their interpretation.
9. Pathway analysis > Why were only model 3 metabolites used for pathway analysis? It is advised to provide data including p-value and the impact score of the pathways identified using the Metaboanalyst. This will give reader a clear picture of how the pathways are significant.
10. Section: Results > Para: Correlations between metabolites and kidney function & Section: Discussion > line 239 and 240:- The authors have mentioned the word “correlation” multiple times. However, in some places, the reader is confused whether it should be correlation or regulation? Also, its is advisable to author to explain how are the “correlating” the metabolites with eGFR are identified.
11. Line 203 > Define CV.
12. Line 234 – 236 > The authors have mentioned the use of two stage design having stringent multiplicity testing and class-based analyses. However it is not well described regarding what are the class in class-based analyses and multiplicity testing.
13. In some table foot notes, authors have mentioned independent variables, it is advisable to mention dependent also for clarity.
14. Table 5 > Probable the authors missed writing “Negatively correlated metabolites”.
15. Table 3 & 5 > Please replace “,” with “.” (coma with decimals) in the values to keep it consistent.
