# Supplementary material for: Impact of kidney function on the metabolome in the general population
Source: PLoS One. 2026 Jun 30;21(6):e0347652. doi: 10.1371/journal.pone.0347652 (PMC13318015; doi:10.1371/journal.pone.0347652)
Supplement: Attachment — Submitted filename: Answers to reviewers_final.docx [file pone.0347652.s008.docx]

**Manuscript: PONE-D-25-35075**

**Title:** Impact of kidney function on the metabolome in the general population

Dear Editor,

We thank you for the thorough review of our manuscript. We have revised the manuscript in accordance with the reviewers’ comments, and we feel it has improved substantially as a result. We herein provide a detailed point-by-point response to the reviewers’ comments. We hope you now will find it suitable for publication in *PLOS One*.

Yours sincerely,

Maria K Svensson MD, PhD

All comments by reviewers are in BLACK font. All responses reviewer’s comments are in RED font.

**Reviewer 1:**

1. **Comment:** From an analytical standpoint, the central variable in this study (eGFR) is derived from creatinine, which is itself a metabolite strongly influenced by muscle mass, diet, and metabolic state. In the discovery cohort, creatinine is not directly measured clinically but instead estimated from metabolomics data using a calibration derived from other cohorts. This creates a dependency structure that can amplify associations and complicates interpretation, particularly for metabolites correlated with creatinine or related metabolic pathways. The manuscript would benefit from a more explicit discussion of this issue and from quantitative validation of the creatinine calibration (e.g., accuracy, bias, and uncertainty). Sensitivity analyses restricted to cohorts with clinically measured creatinine would strengthen confidence in the conclusions.

**Response:** Thank you for this thoughtful and important comment. We agree that it estimating creatinine from metabolomics data in the discovery cohort (EpiHealth) is not optimal compared to direct clinical measurement, and we now explicitly discuss this limitation in the revised manuscript (Discussion section).

Importantly, all statistically significant findings from the discovery cohort were required to replicate in an independent validation cohort in which creatinine was measured using standard clinical chemistry methods. Because the validation analyses are fully independent of the metabolomics-based creatinine calibration, any associations driven by calibration artifacts in the discovery phase would not be expected to replicate. We have now clarified this in the revised manuscript.

In addition, to address the concern that creatinine levels are influenced by muscle mass, diet, and metabolic state, we have now adjusted for related covariates, (energy intake, protein intake, and non-fat mass), in the discovery analyses, thereby reducing potential confounding from these sources (see also Response to Comment 3).

**Change:** Lines 475 - 483

2. **Comment:** Many of the reported associations likely reflect a combination of biological effects and analytical consequences of reduced renal clearance rather than distinct metabolic regulation. While this does not diminish the relevance of the findings, the manuscript should be careful not to imply causal or mechanistic interpretations where the data support association only. Framing the results in terms of metabolite accumulation, clearance, and confounding structure would better align with an analytical chemistry perspective.

**Response:** Thank you for this valuable and insightful comment. We agree that reduced renal clearance can produce widespread metabolite accumulation, which reflects altered elimination rather than distinct metabolic dysregulation. We also agree that our interpretations should not imply causal or mechanistic conclusions where the data support association only.

In response, we have revised the Discussion section to more explicitly frame the observed metabolite changes in terms of accumulation and clearance rather than pathway-specific dysregulation. We now emphasize that, given the predominantly mild reduction in kidney function among participants in these cohorts, the most reasonable explanation for many associations is reduced renal excretion leading to metabolite retention. We hope that this successfully frames the results in a more accurate way.

We have also clarified that our findings are associative and do not establish causal mechanisms. The revised text avoids mechanistic language and instead focuses on patterns consistent with altered clearance.

**Change:** Lines 487-491

3. **Comment:** Adjustment for fat mass and major cardiovascular risk factors is appropriate and strengthens the analysis. However, creatinine-based metrics remain sensitive to lean mass and protein intake, neither of which are fully captured by the covariates used. Residual confounding related to diet, medication use (notably steroids), and sampling conditions is likely and should be acknowledged more clearly, especially given the strong associations observed for steroid-related metabolites.

**Response:** Thank you for this constructive comment. We agree that although adjustment for fat mass and major cardiovascular risk factors strengthens the analysis, creatinine-based metrics remain sensitive to lean mass and protein intake, and that residual confounding from diet, medication use, and sampling conditions warrants careful consideration. In response, we have expanded the set of covariates in the discovery cohort to include protein intake, total energy intake, and fat-free mass (as a proxy for lean mass).

The full discovery analysis was re-run using this extended adjustment model, and all downstream analyses were subsequently updated accordingly. Following this additional adjustment, the number of statistically significant metabolites in the final linear regression model decreased modestly from 317 to 312. This limited change suggests that the overall findings are robust to additional adjustment for dietary intake and lean mass, although some attenuation of associations was observed.

All tables, figures, and corresponding text have been updated to reflect these revised results. Regarding medication use, including corticosteroids, we agree that this is an important potential source of residual confounding, particularly given the strong associations observed for steroid-related metabolites. Unfortunately, detailed medication data were not available for the cohorts included in this study.

We have now explicitly acknowledged this as a limitation in the Discussion section and clarified that unmeasured medication use and other pre-analytical factors (e.g., sampling conditions) may contribute to residual confounding. We were unable to obtain data for medication usage for the participants of the cohorts used in this report. This has been added to the limitations part of the Discussion section.

**Change:** Abstract; Methods section (discovery cohort); Results section; all tables and figures; and the Discussion.

4. **Comment:** The pathway enrichment results are plausible but appear exploratory, as they rely on nominal significance thresholds without clear correction at the pathway level. This is acceptable if explicitly stated, but the manuscript should avoid over-interpreting these findings as definitive biological pathway alterations rather than descriptive clustering of correlated metabolites.

**Response:** Thank you for this important comment. We agree that the pathway enrichment analyses are exploratory in nature and that, given the use of nominal significance thresholds without formal correction at the pathway level, the results should not be interpreted as definitive evidence of biological pathway alterations.

In response, we have revised the manuscript to explicitly state that the pathway enrichment analysis is exploratory and primarily intended to provide descriptive insight into clustering patterns among correlated metabolites. We have also moderated the language in the Results and Discussion section to avoid mechanistic or causal interpretations and instead frame the findings as hypothesis-generating. We believe this clarification ensures that the pathway results are presented with appropriate caution.

**Change:** Line 449 (and related interpretative wording adjusted accordingly).

5. **Comment:** A brief schematic of the analytical workflow (cohorts, models, discovery/validation steps) would improve accessibility for non-specialists

**Response:** We thank the reviewer for this helpful suggestion and we have now added a schematic overview of the analytical workflow. The figure illustrates cohort inclusion, derivation of the exposure variable, covariate adjustment, discovery analyses, and the independent validation step, thereby providing a structured overview of the study design and analytical sequence. We believe this addition substantially improves readability and transparency of the analytical framework and the work in this study.

**Change:** Figure 1; Line 328

**Reviewer 2**

1. **Comment:** The study design is correct by the parameters analysed look outdated. Why was creatinine used for conservative eGDF calculation? Why not kynurenine or serotonin or cystatin C?

**Response:** Thank you for this comment. Creatinine was chosen for eGFR estimation for several reasons. First, data for alternative markers such as cystatin C, kynurenine, or serotonin were not available across the cohorts included in this study. Second, the cohorts were originally established for cardiovascular research, and creatinine remains the most commonly used and clinically validated measure for estimating eGFR in such populations. We have added a brief explanation of this rationale to the manuscript to clarify why creatinine was used and to contextualize the choice within standard clinical practice.

**Change:** Lines 484 – 486

2. **Comment**: The implementation of creatine as a basis for eGFR if further posing a possible inflatory effect. As I understand the cohort setting EpiHealth creatinine is derived from the metabolomics signal via a regression calibrated in POEM/PIVUS80.

**Response:** We thank the reviewer for raising this important point. We acknowledge that estimating creatinine from metabolomics data in the EpiHealth cohort is suboptimal compared with direct clinical measurement. We have added a discussion of this limitation to the revised manuscript (Lines 475–483).

Importantly, all associations identified in the discovery cohort were subsequently tested in an independent validation cohort, in which creatinine was measured using standard clinical chemistry methods. This independent validation effectively mitigates the potential error introduced by metabolomically derived creatinine in the discovery cohort: associations that do not replicate in the validation cohort would not be considered robust.

Regarding the influence of diet and muscle mass on creatinine levels, we have included these variables as covariates in the discovery analyses, which reduces confounding from these sources. *As noted, these points are consistent with our response to the first reviewer comment.*

**Change:** Lines 475 - 483

3. **Comment:** The outcome of the manuscript is largely confirmatory and not novel.

**Response:** We thank the reviewer for this comment. While we acknowledge that several previous studies have examined the relationship between kidney function and metabolomics, we believe our study provides several novel contributions.

First, to our knowledge, this is the largest and most comprehensive population-based study investigating metabolite changes in individuals with only mildly reduced kidney function. Second, our findings demonstrate that metabolite alterations are detectable even at early stages of kidney function decline, whereas most prior studies have focused on patients with moderate to severe kidney impairment.

Thus, the novelty of our study lies in its scale and the ability to capture subtle metabolic changes associated with mild kidney function decline, providing important insights into early kidney-associated metabolic alterations.

4. **Comment:** There is no mechanistic analysis, no causal analyses like Mendelian Radomisation to deconvolute the role(s) of other metabolites changed significantly in relation to eGFR.

**Response:** We thank the reviewer for this important suggestion. In response, we have therefore now added multivariable Mendelian randomization (MVMR) analyses to the manuscript to explore potential causal relationships among metabolites significantly associated with eGFR. The MVMR analyses were adjusted for body mass index (BMI) and type 2 diabetes and were performed on the set of metabolites identified as significant in Model 3.

These analyses have been integrated into the Methods, Results, and Discussion sections, and corresponding tables and figures (Tables 6–7, Figures 1–2) have been updated accordingly. We believe that including MVMR adds a causal perspective to the study and strengthens the interpretation of metabolite-eGFR relationships while still acknowledging the observational nature of the data.

**Change:** Lines 45–52, 95–97, 122–128, 207–259, 283–305, 375–391; majority of the Discussion section; Tables 6–7; Figures 1–2.

**Reviewer 3**

1. **Comment:** Line 108 > please mention degree Celsius or degree Fahrenheit.

**Response:** We thank the reviewer for this comment. The manuscript now explicitly specifies that the temperature is reported in degrees Celsius.

**Changed:** line 139

2. **Comment:** Line 109 > The authors have mentioned the addition of internal standards in plasma samples before extraction. However, the author’s should mention what are the internal standards, their name, their concentration added and how they were useful in the analysis. These details are lacking in the entire manuscript. Also please add them in the results section.

**Response:** Thank you for this important comment. We agree that providing details on the internal standards would strengthen the manuscript. However, and unfortunately, the metabolomic measurements were performed by Metabolon Inc. U.S.A., and certain experimental details, including the specific internal standards and their concentrations, are proprietary and not disclosed to us.

To address the reviewer’s concern, we have updated the Methods section to include all available information regarding the metabolomics workflow. Specifically, we describe the use of pooled matrix samples (obtained by combining small portions of each experimental sample or using well-characterized samples) as technical quality controls, as well as water extractions as negative controls. While the identities and concentrations of the internal standards are not available, we have clearly stated this limitation in the Methods and Results sections to ensure transparency.

**Change:** Lines 137 – 171; Lines 492 – 494.

3. **Comment:** In subjects and methods, the authors have just mentioned the use of reverse phase UPLC-MS/MS and HILIC analytical method for metabolomic study. However, they haven’t mentioned any details, The authors are advised to add details such as column used, mobile phase used, its elution profile, the mass spectrometry parameters, what instrument was the samples analyzed upon. These details are very crucial in determining the overall process of metabolomic analysis, be it targeted or untargeted.

**Response:** We thank the reviewer this comment. We agree that additional methodological details regarding the UPLC-MS/MS and HILIC analyses would strengthen the manuscript. However, as with the previous comment, these experiments were conducted by Metabolon Inc., U.S.A., and detailed information regarding columns, mobile phases, elution profiles, mass spectrometry parameters, and instruments is proprietary and not publicly available.

We have updated the Methods section to clarify that the metabolomic measurements were performed externally and to transparently acknowledge the limitations in reporting specific experimental parameters.

**Change:** Lines 137 – 171; Lines 492 – 494.

4. **Comment:** The authors are advised to provide step by step procedure for conducting experimental metabolomic analysis.

**Response:** We thank the reviewer for this comment. As noted in our responses to the previous two comments, the metabolomic analyses were performed by Metabolon Inc., U.S.A., and the step-by-step experimental procedures are proprietary and have not been made publicly available.

Consequently, we are unable to provide a detailed experimental protocol. We have clarified this limitation in the Methods section to ensure transparency regarding the external data generation.

**Change:** Lines 137 – 171; Lines 492 – 494.

5. **Comment:** The authors are also advised to present steps of identification of metabolites from untargeted to targeted, their selection and annotation criteria, and software used in this process.

**Response:** Thank you for this comment. We have added a section to the method section adressing this. In brief, the identification was done by comparing the retention indes, mass to charge ratio and cromatographic data from the experiment with a library with >3,000 metabolites which metabolon maintains.

**Change:** Lines 137 – 171; Lines 492 – 494.

6. **Comment:** The authors have mentioned the samples from all three cohorts were analyzed in same fashion and calibrated for being comparable. Please clarify how they were calibrated.

**Response:** We thank the reviewer for this comment. We have added a section to the Methods addressing the metabolite identification and annotation process. In brief, metabolites were identified by comparing retention indices, mass-to-charge ratios, and chromatographic data from the experiments against a proprietary reference library maintained by Metabolon Inc., which includes over 3,000 metabolites. This approach allowed for confident annotation and selection of metabolites for downstream analyses. Please also refer to our previous responses to comment 2-4.

**Change:** Lines 137 – 171; Lines 492 – 494.

7. **Comment:** Multivariate analysis is mentioned in the abstract. However, what multivariate analysis, like PCA, or PLSDA or any other analysis was used and their results are missing in the manuscript. Please clarify how the multivariate analysis was used.

**Response:** We thank the reviewer for pointing this out. This was an error on our part. This was an oversight in terminology: we did not perform multivariate analyses (e.g., PCA or PLS-DA). Instead, all analyses were *multivariable*, specifically multivariable linear regression models. All instances of “multivariate analysis” in the manuscript have been corrected to “multivariable analysis” to accurately reflect the statistical approach used.

**Change:**  All instances of the term “multivariate” corrected to “multivariable.”

8. **Comment:** Please explain in detail what the validation models are and clarify their interpretation.

**Response:** We thank the reviewer for this comment. We have revised the Methods section to clarify the discovery and validation analyses. The validation models are linear regression models in which the *rank-normalized metabolite concentration* (measured in arbitrary units) serves as the dependent variable. The independent variables vary depending on the specific model. The interpretation of these models is that the rank-normalized metabolite concentration can be expressed as a linear combination of the independent variables, each weighted by its corresponding regression coefficient.

**Change:** Lines 265-267

9. **Comment:** Pathway analysis > Why were only model 3 metabolites used for pathway analysis? It is advised to provide data including p-value and the impact score of the pathways identified using the Metaboanalyst. This will give reader a clear picture of how the pathways are significant.

**Response:** We thank the reviewer for this comment. In the original analysis, only metabolites from Model 3 (the fully adjusted model) were used for pathway enrichment because this model represented the primary findings of the study. In the updated manuscript, following the addition of multivariable Mendelian randomization analyses, metabolites identified through this causal framework are now used for pathway enrichment, as they represent the main results of the report. We have also added the ***pathway impact scores*** and p-values in Table 7, providing readers with a clearer picture of pathway significance and relative influence.

**Change:** Table 7

10. **Comment:** Section: Results > Para: Correlations between metabolites and kidney function & Section: Discussion > line 239 and 240:- The authors have mentioned the word “correlation” multiple times. However, in some places, the reader is confused whether it should be correlation or regulation? Also, its is advisable to author to explain how are the “correlating” the metabolites with eGFR are identified.

**Response:** We thank the reviewer for this comment. In the manuscript, the term “correlation” was originally used to refer to the regression coefficient for each metabolite. To avoid confusion with mechanistic regulation, all instances of ***correlation, correlated, correlating, or correlate*** referring to our analyses have been replaced with ***association*** or ***regression coefficient***, as appropriate. This clarifies that the reported relationships are statistical associations rather than implying causal or regulatory mechanisms.

**Changed:**  All instances of the terms correlation, correlated, correlating, or correlate, referring to analyses performed in the Results and Discussion sections.

11. **Comment:** Line 203 > Define CV.

**Response:** We thank the reviewer for this comment. The abbreviation **CV** has been replaced with ***cardiovascular*** to ensure clarity.

**Change:** line 356

12. **Comment:** Line 234 – 236 > The authors have mentioned the use of two stage design having stringent multiplicity testing and class-based analyses. However, it is not well described regarding what are the class in class-based analyses and multiplicity testing.

**Response:** We thank the reviewer for pointing this out. We acknowledge that the description of “class-based analyses of metabolites” was unclear. To avoid confusion, we have removed the phrase **“*and class-based analyses of metabolites*”** from the manuscript.

**Change:** Lines 397

13. **Comment:** In some table foot notes, authors have mentioned independent variables, it is advisable to mention dependent also for clarity.

**Response:** The dependent variables have been added to these sections.

**Change:** Below table 2-5

14. **Comment:** Table 5 > Probable the authors missed writing “Negatively correlated metabolites”.

**Response:** We added the term “metabolites with negative regression coefficients” to that section.

**Change:** Table 5

15. **Comment:** Table 3 & 5 > Please replace “,” with “.” (coma with decimals) in the values to keep it consistent.

**Response:** All decimal commas have been changed to decimal points in the tables.

**Change**: All tables in the report.
