# Supplementary material for: Impact of kidney function on the metabolome in the general population
Source: PLoS One. 2026 Jun 30;21(6):e0347652. doi: 10.1371/journal.pone.0347652 (PMC13318015; doi:10.1371/journal.pone.0347652)
Supplement: Peer review history

# Peer review: Decision Letter 0

**DOI:** 10.1371/journal.pone.0347652.r001

**Authors:** Anil Bhatia

5 Jan 2026

PONE-D-25-35075

Impact of kidney function on the metabolome in the general population

PLOS One

Dear Dr. Svensson,

Thank you for submitting your manuscript to PLOS ONE. After careful consideration, we feel that it has merit but does not fully meet PLOS ONE’s publication criteria as it currently stands. Therefore, we invite you to submit a revised version of the manuscript that addresses the points raised during the review process.

Please submit your revised manuscript by Feb 19 2026 11:59PM. If you will need more time than this to complete your revisions, please reply to this message or contact the journal office at plosone@plos.org. When you're ready to submit your revision, log on to https://www.editorialmanager.com/pone/ and select the 'Submissions Needing Revision' folder to locate your manuscript file.

Please include the following items when submitting your revised manuscript:

- A letter that responds to each point raised by the academic editor and reviewer(s). You should upload this letter as a separate file labeled 'Response to Reviewers'.
- A marked-up copy of your manuscript that highlights changes made to the original version. You should upload this as a separate file labeled 'Revised Manuscript with Track Changes'.
- An unmarked version of your revised paper without tracked changes. You should upload this as a separate file labeled 'Manuscript'.

If you would like to make changes to your financial disclosure, please include your updated statement in your cover letter. Guidelines for resubmitting your figure files are available below the reviewer comments at the end of this letter.

If applicable, we recommend that you deposit your laboratory protocols in protocols.io to enhance the reproducibility of your results. Protocols.io assigns your protocol its own identifier (DOI) so that it can be cited independently in the future. For instructions see: https://journals.plos.org/plosone/s/submission-guidelines#loc-laboratory-protocols. Additionally, PLOS ONE offers an option for publishing peer-reviewed Lab Protocol articles, which describe protocols hosted on protocols.io. Read more information on sharing protocols at https://plos.org/protocols?utm_medium=editorial-email&utm_source=authorletters&utm_campaign=protocols.

We look forward to receiving your revised manuscript.

Kind regards,

Anil Bhatia, Ph.D

Academic Editor

PLOS One

**Journal Requirements:**

When submitting your revision, we need you to address these additional requirements.

1. Please ensure that your manuscript meets PLOS ONE's style requirements, including those for file naming. The PLOS ONE style templates can be found at

https://journals.plos.org/plosone/s/file?id=wjVg/PLOSOne_formatting_sample_main_body.pdf and

https://journals.plos.org/plosone/s/file?id=ba62/PLOSOne_formatting_sample_title_authors_affiliations.pdf

2. Please provide additional details regarding participant consent. In the ethics statement in the Methods and online submission information, please ensure that you have specified what type you obtained (for instance, written or verbal, and if verbal, how it was documented and witnessed). If your study included minors, state whether you obtained consent from parents or guardians. If the need for consent was waived by the ethics committee, please include this information.

3. We note that the grant information you provided in the ‘Funding Information’ and ‘Financial Disclosure’ sections do not match.

When you resubmit, please ensure that you provide the correct grant numbers for the awards you received for your study in the ‘Funding Information’ section.

4. Thank you for stating the following financial disclosure:

“The EpiHealth study is funded as a strategic research area by the Swedish government. PIVUS80 and POEM were funded by Uppsala University Hospital. In addition, the Uppsala University Hospital ALF grants and establishment funding (MKS), Swedish National Strategic Research Initiative EXODIAB (Excellence of Diabetes Research in Sweden), the Swedish Kidney Foundation (Njurfonden)  and the Family Ernfors Foundation funded this study.”

Please state what role the funders took in the study.  If the funders had no role, please state: "The funders had no role in study design, data collection and analysis, decision to publish, or preparation of the manuscript."

If this statement is not correct you must amend it as needed.

Please include this amended Role of Funder statement in your cover letter; we will change the online submission form on your behalf.

5. Please note that funding information should not appear in any section or other areas of your manuscript. We will only publish funding information present in the Funding Statement section of the online submission form. Please remove any funding-related text from the manuscript.

6. In the online submission form you indicate that your data is not available for proprietary reasons and have provided a contact point for accessing this data. Please note that your current contact point is a co-author on this manuscript. According to our Data Policy, the contact point must not be an author on the manuscript and must be an institutional contact, ideally not an individual. Please revise your data statement to a non-author institutional point of contact, such as a data access or ethics committee, and send this to us via return email. Please also include contact information for the third party organization, and please include the full citation of where the data can be found.

7. We note that you have included the phrase “data not shown” in your manuscript. Unfortunately, this does not meet our data sharing requirements. PLOS does not permit references to inaccessible data. We require that authors provide all relevant data within the paper, Supporting Information files, or in an acceptable, public repository. Please add a citation to support this phrase or upload the data that corresponds with these findings to a stable repository (such as Figshare or Dryad) and provide and URLs, DOIs, or accession numbers that may be used to access these data. Or, if the data are not a core part of the research being presented in your study, we ask that you remove the phrase that refers to these data.

8. Please include captions for your Supporting Information files at the end of your manuscript, and update any in-text citations to match accordingly. Please see our Supporting Information guidelines for more information: http://journals.plos.org/plosone/s/supporting-information.

9. If the reviewer comments include a recommendation to cite specific previously published works, please review and evaluate these publications to determine whether they are relevant and should be cited. There is no requirement to cite these works unless the editor has indicated otherwise.

[Note: HTML markup is below. Please do not edit.]

Reviewers' comments:

Reviewer's Responses to Questions

**Comments to the Author**

1. Is the manuscript technically sound, and do the data support the conclusions?

The manuscript must describe a technically sound piece of scientific research with data that supports the conclusions. Experiments must have been conducted rigorously, with appropriate controls, replication, and sample sizes. The conclusions must be drawn appropriately based on the data presented.

Reviewer #1: Yes

Reviewer #2: No

Reviewer #3: No

**********

2. Has the statistical analysis been performed appropriately and rigorously?

Reviewer #1: I Don't Know

Reviewer #2: No

Reviewer #3: No

**********

3. Have the authors made all data underlying the findings in their manuscript fully available?

The PLOS Data policy requires authors to make all data underlying the findings described in their manuscript fully available without restriction, with rare exception (please refer to the Data Availability Statement in the manuscript PDF file). The data should be provided as part of the manuscript or its supporting information, or deposited to a public repository. For example, in addition to summary statistics, the data points behind means, medians and variance measures should be available. If there are restrictions on publicly sharing data—e.g. participant privacy or use of data from a third party—those must be specified.

Reviewer #1: Yes

Reviewer #2: Yes

Reviewer #3: No

**********

4. Is the manuscript presented in an intelligible fashion and written in standard English?

PLOS ONE does not copyedit accepted manuscripts, so the language in submitted articles must be clear, correct, and unambiguous. Any typographical or grammatical errors should be corrected at revision, so please note any specific errors here.

Reviewer #1: Yes

Reviewer #2: Yes

Reviewer #3: No

**********

5. Review Comments to the Author

Please use the space provided to explain your answers to the questions above. You may also include additional comments for the author, including concerns about dual publication, research ethics, or publication ethics. (Please upload your review as an attachment if it exceeds 20,000 characters)

Reviewer #1: This is a well-executed population-scale metabolomics study that demonstrates widespread associations between kidney function and the circulating metabolome in the general population. The use of multiple cohorts with a discovery validation framework is a major strength, and the findings reinforce an important analytical point: kidney function must be carefully considered when interpreting metabolomics data, even outside overt chronic kidney disease. While many of the observed associations are expected from an analytical chemistry perspective, the scale and consistency of the results give the work value. However, several methodological and interpretational issues, particularly related to the use of creatinine-based eGFR and derived variables, need to be addressed to ensure the conclusions are technically sound and appropriately framed.

Major revision:

1) From an analytical standpoint, the central variable in this study (eGFR) is derived from creatinine, which is itself a metabolite strongly influenced by muscle mass, diet, and metabolic state. In the discovery cohort, creatinine is not directly measured clinically but instead estimated from metabolomics data using a calibration derived from other cohorts. This creates a dependency structure that can amplify associations and complicates interpretation, particularly for metabolites correlated with creatinine or related metabolic pathways. The manuscript would benefit from a more explicit discussion of this issue and from quantitative validation of the creatinine calibration (e.g., accuracy, bias, and uncertainty). Sensitivity analyses restricted to cohorts with clinically measured creatinine would strengthen confidence in the conclusions.

2) Many of the reported associations likely reflect a combination of biological effects and analytical consequences of reduced renal clearance rather than distinct metabolic regulation. While this does not diminish the relevance of the findings, the manuscript should be careful not to imply causal or mechanistic interpretations where the data support association only. Framing the results in terms of metabolite accumulation, clearance, and confounding structure would better align with an analytical chemistry perspective.

3) Adjustment for fat mass and major cardiovascular risk factors is appropriate and strengthens the analysis. However, creatinine-based metrics remain sensitive to lean mass and protein intake, neither of which are fully captured by the covariates used. Residual confounding related to diet, medication use (notably steroids), and sampling conditions is likely and should be acknowledged more clearly, especially given the strong associations observed for steroid-related metabolites.

4) The pathway enrichment results are plausible but appear exploratory, as they rely on nominal significance thresholds without clear correction at the pathway level. This is acceptable if explicitly stated, but the manuscript should avoid over-interpreting these findings as definitive biological pathway alterations rather than descriptive clustering of correlated metabolites.

Minor revisions:

1) A brief schematic of the analytical workflow (cohorts, models, discovery/validation steps) would improve accessibility for non-specialists

Reviewer #2: 1. The study design is correct by the parameters analysed look outdated. Why was creatinine used for conservative eGDF calculation? Why not kynurenine or serotonin or cystatin C?

2. The implementation of creatine as a basis for eGFR if further posing a possible inflatory effect. As I understand the cohort setting EpiHealth creatinine is derrived from the metabolomics signal via a regression calibrated in POEM/PIVUS80.

3. The outcome of the manuscript is largely confirmatory and not novel.

4. There is no mechanistic analysis, no causal analyses like Mendelian Radomisation to deconvolute the role(s) of other metabolites changed significantly in relation to eGFR.

Reviewer #3: The current manuscript provides a timely and comprehensive metabolomic investigation into the associations between estimated glomerular filtration rate (eGFR) and a wide array of endogenous metabolites in a large, general population cohort. The research directly addresses an important pathophysiological hypothesis—that retained metabolites may mediate the well-established link between chronic kidney disease and cardiovascular risk.

However, there are following questions that should be answered/clarified by the author and updated in the manuscript that will help in overall quality of the manuscript.

1. Line 108 > please mention degree Celsius or degree Fahrenheit.

2. Line 109 > The authors have mentioned the addition of internal standards in plasma samples before extraction. However, the author’s should mention what are the internal standards, their name, their concentration added and how they were useful in the analysis. These details are lacking in the entire manuscript. Also please add them in the results section.

3. In subjects and methods, the authors have just mentioned the use of reverse phase UPLC-MS/MS and HILIC analytical method for metabolomic study. However, they haven’t mentioned any details, The authors are advised to add details such as column used, mobile phase used, its elution profile, the mass spectrometry parameters, what instrument was the samples analyzed upon. These details are very crucial in determining the overall process of metabolomic analysis, be it targeted or untargeted.

4. The authors are advised to provide step by step procedure for conducting experimental metabolomic analysis.

5. The authors are also advised to present steps of identification of metabolites from untargeted to targeted, their selection and annotation criteria, and software used in this process.

6. The authors have mentioned the samples from all three cohorts were analyzed in same fashion and calibrated for being comparable. Please clarify how they were calibrated.

7. Multivariate analysis is mentioned in the abstract. However, what multivariate analysis, like PCA, or PLSDA or any other analysis was used and their results are missing in the manuscript. Please clarify how the multivariate analysis was used.

8. Please explain in detail what the validation models are and clarify their interpretation.

9. Pathway analysis > Why were only model 3 metabolites used for pathway analysis? It is advised to provide data including p-value and the impact score of the pathways identified using the Metaboanalyst. This will give reader a clear picture of how the pathways are significant.

10. Section: Results > Para: Correlations between metabolites and kidney function & Section: Discussion > line 239 and 240:- The authors have mentioned the word “correlation” multiple times. However, in some places, the reader is confused whether it should be correlation or regulation? Also, its is advisable to author to explain how are the “correlating” the metabolites with eGFR are identified.

11. Line 203 > Define CV.

12. Line 234 – 236 > The authors have mentioned the use of two stage design having stringent multiplicity testing and class-based analyses. However it is not well described regarding what are the class in class-based analyses and multiplicity testing.

13. In some table foot notes, authors have mentioned independent variables, it is advisable to mention dependent also for clarity.

14. Table 5 > Probable the authors missed writing “Negatively correlated metabolites”.

15. Table 3 & 5 > Please replace “,” with “.” (coma with decimals) in the values to keep it consistent.

**********

6. PLOS authors have the option to publish the peer review history of their article (what does this mean?). If published, this will include your full peer review and any attached files.

If you choose “no”, your identity will remain anonymous but your review may still be made public.

**Do you want your identity to be public for this peer review?**  For information about this choice, including consent withdrawal, please see our Privacy Policy.

Reviewer #1: No

Reviewer #2: No

Reviewer #3: No

**********

[NOTE: If reviewer comments were submitted as an attachment file, they will be attached to this email and accessible via the submission site. Please log into your account, locate the manuscript record, and check for the action link "View Attachments". If this link does not appear, there are no attachment files.]

To ensure your figures meet our technical requirements, please review our figure guidelines: https://journals.plos.org/plosone/s/figures

You may also use PLOS’s free figure tool, NAAS, to help you prepare publication quality figures: https://journals.plos.org/plosone/s/figures#loc-tools-for-figure-preparation.

NAAS will assess whether your figures meet our technical requirements by comparing each figure against our figure specifications.

Attachment Submitted filename: Reviewer comments.docx

# Author comment: Author response to Decision Letter 1

**DOI:** 10.1371/journal.pone.0347652.r002

23 Mar 2026

Manuscript: PONE-D-25-35075

Title: Impact of kidney function on the metabolome in the general population

Dear Editor,

We thank you for the thorough review of our manuscript. We have revised the manuscript in accordance with the reviewers’ comments, and we feel it has improved substantially as a result. We herein provide a detailed point-by-point response to the reviewers’ comments. We hope you now will find it suitable for publication in PLOS One.

Yours sincerely,

Maria K Svensson MD, PhD

All comments by reviewers are in BLACK font. All responses reviewer’s comments are in RED font.

Reviewer 1:

1. Comment: From an analytical standpoint, the central variable in this study (eGFR) is derived from creatinine, which is itself a metabolite strongly influenced by muscle mass, diet, and metabolic state. In the discovery cohort, creatinine is not directly measured clinically but instead estimated from metabolomics data using a calibration derived from other cohorts. This creates a dependency structure that can amplify associations and complicates interpretation, particularly for metabolites correlated with creatinine or related metabolic pathways. The manuscript would benefit from a more explicit discussion of this issue and from quantitative validation of the creatinine calibration (e.g., accuracy, bias, and uncertainty). Sensitivity analyses restricted to cohorts with clinically measured creatinine would strengthen confidence in the conclusions.

Response: Thank you for this thoughtful and important comment. We agree that it estimating creatinine from metabolomics data in the discovery cohort (EpiHealth) is not optimal compared to direct clinical measurement, and we now explicitly discuss this limitation in the revised manuscript (Discussion section).

Importantly, all statistically significant findings from the discovery cohort were required to replicate in an independent validation cohort in which creatinine was measured using standard clinical chemistry methods. Because the validation analyses are fully independent of the metabolomics-based creatinine calibration, any associations driven by calibration artifacts in the discovery phase would not be expected to replicate. We have now clarified this in the revised manuscript.

In addition, to address the concern that creatinine levels are influenced by muscle mass, diet, and metabolic state, we have now adjusted for related covariates, (energy intake, protein intake, and non-fat mass), in the discovery analyses, thereby reducing potential confounding from these sources (see also Response to Comment 3).

Change: Lines 475 - 483

2. Comment: Many of the reported associations likely reflect a combination of biological effects and analytical consequences of reduced renal clearance rather than distinct metabolic regulation. While this does not diminish the relevance of the findings, the manuscript should be careful not to imply causal or mechanistic interpretations where the data support association only. Framing the results in terms of metabolite accumulation, clearance, and confounding structure would better align with an analytical chemistry perspective.

Response: Thank you for this valuable and insightful comment. We agree that reduced renal clearance can produce widespread metabolite accumulation, which reflects altered elimination rather than distinct metabolic dysregulation. We also agree that our interpretations should not imply causal or mechanistic conclusions where the data support association only.

In response, we have revised the Discussion section to more explicitly frame the observed metabolite changes in terms of accumulation and clearance rather than pathway-specific dysregulation. We now emphasize that, given the predominantly mild reduction in kidney function among participants in these cohorts, the most reasonable explanation for many associations is reduced renal excretion leading to metabolite retention. We hope that this successfully frames the results in a more accurate way.

We have also clarified that our findings are associative and do not establish causal mechanisms. The revised text avoids mechanistic language and instead focuses on patterns consistent with altered clearance.

Change: Lines 487-491

3. Comment: Adjustment for fat mass and major cardiovascular risk factors is appropriate and strengthens the analysis. However, creatinine-based metrics remain sensitive to lean mass and protein intake, neither of which are fully captured by the covariates used. Residual confounding related to diet, medication use (notably steroids), and sampling conditions is likely and should be acknowledged more clearly, especially given the strong associations observed for steroid-related metabolites.

Response: Thank you for this constructive comment. We agree that although adjustment for fat mass and major cardiovascular risk factors strengthens the analysis, creatinine-based metrics remain sensitive to lean mass and protein intake, and that residual confounding from diet, medication use, and sampling conditions warrants careful consideration. In response, we have expanded the set of covariates in the discovery cohort to include protein intake, total energy intake, and fat-free mass (as a proxy for lean mass).

The full discovery analysis was re-run using this extended adjustment model, and all downstream analyses were subsequently updated accordingly. Following this additional adjustment, the number of statistically significant metabolites in the final linear regression model decreased modestly from 317 to 312. This limited change suggests that the overall findings are robust to additional adjustment for dietary intake and lean mass, although some attenuation of associations was observed.

All tables, figures, and corresponding text have been updated to reflect these revised results. Regarding medication use, including corticosteroids, we agree that this is an important potential source of residual confounding, particularly given the strong associations observed for steroid-related metabolites. Unfortunately, detailed medication data were not available for the cohorts included in this study.

We have now explicitly acknowledged this as a limitation in the Discussion section and clarified that unmeasured medication use and other pre-analytical factors (e.g., sampling conditions) may contribute to residual confounding. We were unable to obtain data for medication usage for the participants of the cohorts used in this report. This has been added to the limitations part of the Discussion section.

Change: Abstract; Methods section (discovery cohort); Results section; all tables and figures; and the Discussion.

4. Comment: The pathway enrichment results are plausible but appear exploratory, as they rely on nominal significance thresholds without clear correction at the pathway level. This is acceptable if explicitly stated, but the manuscript should avoid over-interpreting these findings as definitive biological pathway alterations rather than descriptive clustering of correlated metabolites.

Response: Thank you for this important comment. We agree that the pathway enrichment analyses are exploratory in nature and that, given the use of nominal significance thresholds without formal correction at the pathway level, the results should not be interpreted as definitive evidence of biological pathway alterations.

In response, we have revised the manuscript to explicitly state that the pathway enrichment analysis is exploratory and primarily intended to provide descriptive insight into clustering patterns among correlated metabolites. We have also moderated the language in the Results and Discussion section to avoid mechanistic or causal interpretations and instead frame the findings as hypothesis-generating. We believe this clarification ensures that the pathway results are presented with appropriate caution.

Change: Line 449 (and related interpretative wording adjusted accordingly).

5. Comment: A brief schematic of the analytical workflow (cohorts, models, discovery/validation steps) would improve accessibility for non-specialists

Response: We thank the reviewer for this helpful suggestion and we have now added a schematic overview of the analytical workflow. The figure illustrates cohort inclusion, derivation of the exposure variable, covariate adjustment, discovery analyses, and the independent validation step, thereby providing a structured overview of the study design and analytical sequence. We believe this addition substantially improves readability and transparency of the analytical framework and the work in this study.

Change: Figure 1; Line 328

Reviewer 2

1. Comment: The study design is correct by the parameters analysed look outdated. Why was creatinine used for conservative eGDF calculation? Why not kynurenine or serotonin or cystatin C?

Response: Thank you for this comment. Creatinine was chosen for eGFR estimation for several reasons. First, data for alternative markers such as cystatin C, kynurenine, or serotonin were not available across the cohorts included in this study. Second, the cohorts were originally established for cardiovascular research, and creatinine remains the most commonly used and clinically validated measure for estimating eGFR in such populations. We have added a brief explanation of this rationale to the manuscript to clarify why creatinine was used and to contextualize the choice within standard clinical practice.

Change: Lines 484 – 486

2. Comment: The implementation of creatine as a basis for eGFR if further posing a possible inflatory effect. As I understand the cohort setting EpiHealth creatinine is derived from the metabolomics signal via a regression calibrated in POEM/PIVUS80.

Response: We thank the reviewer for raising this important point. We acknowledge that estimating creatinine from metabolomics data in the EpiHealth cohort is suboptimal compared with direct clinical measurement. We have added a discussion of this limitation to the revised manuscript (Lines 475–483).

Importantly, all associations identified in the discovery cohort were subsequently tested in an independent validation cohort, in which creatinine was measured using standard clinical chemistry methods. This independent validation effectively mitigates the potential error introduced by metabolomically derived creatinine in the discovery cohort: associations that do not replicate in the validation cohort would not be considered robust.

Regarding the influence of diet and muscle mass on creatinine levels, we have included these variables as covariates in the discovery analyses, which reduces confounding from these sources. As noted, these points are consistent with our response to the first reviewer comment.

Change: Lines 475 - 483

3. Comment: The outcome of the manuscript is largely confirmatory and not novel.

Response: We thank the reviewer for this comment. While we acknowledge that several previous studies have examined the relationship between kidney function and metabolomics, we believe our study provides several novel contributions.

First, to our knowledge, this is the largest and most comprehensive population-based study investigating metabolite changes in individuals with only mildly reduced kidney function. Second, our findings demonstrate that metabolite alterations are detectable even at early stages of kidney function decline, whereas most prior studies have focused on patients with moderate to severe kidney impairment.

Thus, the novelty of our study lies in its scale and the ability to capture subtle metabolic changes associated with mild kidney function decline, providing important insights into early kidney-associated metabolic alterations.

4. Comment: There is no mechanistic analysis, no causal analyses like Mendelian Radomisation to deconvolute the role(s) of other metabolites changed significantly in relation to eGFR.

Response: We thank the reviewer for this important suggestion. In response, we have therefore now added multivariable Mendelian randomization (MVMR) analyses to the manuscript to explore potential causal relationships among metabolites significantly associated with eGFR. The MVMR analyses were adjusted for body mass index (BMI) and type 2 diabetes and were performed on the set of metabolites identified as significant in Model 3.

These analyses have been integrated into the Methods, Results, and Discussion sections, and corresponding tables and figures (Tables 6–7, Figures 1–2) have been updated accordingly. We believe that including MVMR adds a causal perspective to the study and strengthens the interpretation of metabolite-eGFR relationships while still acknowledging the observational nature of the data.

Change: Lines 45–52, 95–97, 122–128, 207–259, 283–305, 375–391; majority of the Discussion section; Tables 6–7; Figures 1–2.

Reviewer 3

1. Comment: Line 108 > please mention degree Celsius or degree Fahrenheit.

Response: We thank the reviewer for this comment. The manuscript now explicitly specifies that the temperature is reported in degrees Celsius.

Changed: line 139

2. Comment: Line 109 > The authors have mentioned the addition of internal standards in plasma samples before extraction. However, the author’s should mention what are the internal standards, their name, their concentration added and how they were useful in the analysis. These details are lacking in the entire manuscript. Also please add them in the results section.

Response: Thank you for this important comment. We agree that providing details on the internal standards would strengthen the manuscript. However, and unfortunately, the metabolomic measurements were performed by Metabolon Inc. U.S.A., and certain experimental details, including the specific internal standards and their concentrations, are proprietary and not disclosed to us.

To address the reviewer’s concern, we have updated the Methods section to include all available information regarding the metabolomics workflow. Specifically, we describe the use of pooled matrix samples (obtained by combining small portions of each experimental sample or using well-characterized samples) as technical quality controls, as well as water extractions as negative controls. While the identities and concentrations of the internal standards are not available, we have clearly stated this limitation in the Methods and Results sections to ensure transparency.

Change: Lines 137 – 171; Lines 492 – 494.

3. Comment: In subjects and methods, the authors have just mentioned the use of reverse phase UPLC-MS/MS and HILIC analytical method for metabolomic study. However, they haven’t mentioned any details, The authors are advised to add details such as column used, mobile phase used, its elution profile, the mass spectrometry parameters, what instrument was the samples analyzed upon. These details are very crucial in determining the overall process of metabolomic analysis, be it targeted or untargeted.

Response: We thank the reviewer this comment. We agree that additional methodological details regarding the UPLC-MS/MS and HILIC analyses would strengthen the manuscript. However, as with the previous comment, these experiments were conducted by Metabolon Inc., U.S.A., and detailed information regarding columns, mobile phases, elution profiles, mass spectrometry parameters, and instruments is proprietary and not publicly available.

We have updated the Methods section to clarify that the metabolomic measurements were performed externally and to transparently acknowledge the limitations in reporting specific experimental parameters.

Change: Lines 137 – 171; Lines 492 – 494.

4. Comment: The authors are advised to provide step by step procedure for conducting experimental metabolomic analysis.

Response: We thank the reviewer for this comment. As noted in our responses to the previous two comments, the metabolomic analyses were performed by Metabolon Inc., U.S.A., and the step-by-step experimental procedures are proprietary and have not been made publicly available.

Consequently, we are unable to provide a detailed experimental protocol. We have clarified this limitation in the Methods section to ensure transparency regarding the external data generation.

Change: Lines 137 – 171; Lines 492 – 494.

5. Comment: The a

Attachment Submitted filename: Answers to reviewers_final.docx

# Peer review: Decision Letter 1

**DOI:** 10.1371/journal.pone.0347652.r003

**Authors:** Anil Bhatia

7 Apr 2026

Impact of kidney function on the metabolome in the general population

PONE-D-25-35075R1

Dear Dr. Svensson,

We’re pleased to inform you that your manuscript has been judged scientifically suitable for publication and will be formally accepted for publication once it meets all outstanding technical requirements.

Within one week, you’ll receive an e-mail detailing the required amendments. When these have been addressed, you’ll receive a formal acceptance letter and your manuscript will be scheduled for publication.

An invoice will be generated when your article is formally accepted. Please note, if your institution has a publishing partnership with PLOS and your article meets the relevant criteria, all or part of your publication costs will be covered. Please make sure your user information is up-to-date by logging into Editorial Manager at Editorial Manager® and clicking the ‘Update My Information' link at the top of the page. For questions related to billing, please contact billing support.

If your institution or institutions have a press office, please notify them about your upcoming paper to help maximize its impact. If they’ll be preparing press materials, please inform our press team as soon as possible -- no later than 48 hours after receiving the formal acceptance. Your manuscript will remain under strict press embargo until 2 pm Eastern Time on the date of publication. For more information, please contact onepress@plos.org.

Kind regards,

Anil Bhatia, Ph.D

Academic Editor

PLOS One

Additional Editor Comments (optional):

Reviewers' comments:

Reviewer's Responses to Questions

**Comments to the Author**

1. If the authors have adequately addressed your comments raised in a previous round of review and you feel that this manuscript is now acceptable for publication, you may indicate that here to bypass the “Comments to the Author” section, enter your conflict of interest statement in the “Confidential to Editor” section, and submit your "Accept" recommendation.

Reviewer #1: All comments have been addressed

Reviewer #3: All comments have been addressed

**********

2. Is the manuscript technically sound, and do the data support the conclusions?

The manuscript must describe a technically sound piece of scientific research with data that supports the conclusions. Experiments must have been conducted rigorously, with appropriate controls, replication, and sample sizes. The conclusions must be drawn appropriately based on the data presented.

Reviewer #1: Yes

Reviewer #3: Yes

**********

3. Has the statistical analysis been performed appropriately and rigorously?

Reviewer #1: Yes

Reviewer #3: Yes

**********

4. Have the authors made all data underlying the findings in their manuscript fully available?

The PLOS Data policy requires authors to make all data underlying the findings described in their manuscript fully available without restriction, with rare exception (please refer to the Data Availability Statement in the manuscript PDF file). The data should be provided as part of the manuscript or its supporting information, or deposited to a public repository. For example, in addition to summary statistics, the data points behind means, medians and variance measures should be available. If there are restrictions on publicly sharing data—e.g. participant privacy or use of data from a third party—those must be specified.

Reviewer #1: Yes

Reviewer #3: Yes

**********

5. Is the manuscript presented in an intelligible fashion and written in standard English?

PLOS ONE does not copyedit accepted manuscripts, so the language in submitted articles must be clear, correct, and unambiguous. Any typographical or grammatical errors should be corrected at revision, so please note any specific errors here.

Reviewer #1: Yes

Reviewer #3: Yes

**********

6. Review Comments to the Author

Please use the space provided to explain your answers to the questions above. You may also include additional comments for the author, including concerns about dual publication, research ethics, or publication ethics. (Please upload your review as an attachment if it exceeds 20,000 characters)

Reviewer #1: (No Response)

Reviewer #3: (No Response)

**********

7. PLOS authors have the option to publish the peer review history of their article (what does this mean?). If published, this will include your full peer review and any attached files.

If you choose “no”, your identity will remain anonymous but your review may still be made public.

**Do you want your identity to be public for this peer review?**  For information about this choice, including consent withdrawal, please see our Privacy Policy.

Reviewer #1: No

Reviewer #3: No

**********

# Editor report: Acceptance letter

**DOI:** 10.1371/journal.pone.0347652.r004

**Authors:** Anil Bhatia

PONE-D-25-35075R1

PLOS One

Dear Dr. Svensson,

I'm pleased to inform you that your manuscript has been deemed suitable for publication in PLOS One. Congratulations! Your manuscript is now being handed over to our production team.

At this stage, our production department will prepare your paper for publication. This includes ensuring the following:

* All references, tables, and figures are properly cited

* All relevant supporting information is included in the manuscript submission,

* There are no issues that prevent the paper from being properly typeset

You will receive further instructions from the production team, including instructions on how to review your proof when it is ready. Please keep in mind that we are working through a large volume of accepted articles, so please give us a few days to review your paper and let you know the next and final steps.

Lastly, if your institution or institutions have a press office, please let them know about your upcoming paper now to help maximize its impact. If they'll be preparing press materials, please inform our press team within the next 48 hours. Your manuscript will remain under strict press embargo until 2 pm Eastern Time on the date of publication. For more information, please contact onepress@plos.org.

You will receive an invoice from PLOS for your publication fee after your manuscript has reached the completed accept phase. If you receive an email requesting payment before acceptance or for any other service, this may be a phishing scheme. Learn how to identify phishing emails and protect your accounts at https://explore.plos.org/phishing.

If we can help with anything else, please email us at customercare@plos.org.

Thank you for submitting your work to PLOS ONE and supporting open access.

Kind regards,

PLOS ONE Editorial Office Staff

on behalf of

Dr. Anil Bhatia

Academic Editor

PLOS One
